# ApoE4 Upregulates GSK‐3β to Aggravate Alzheimer‐Like Pathologies and Cognitive Impairment in Type 2 Diabetic Mice

**DOI:** 10.1111/cns.70575

**Published:** 2025-09-04

**Authors:** Yuying Wang, Yang Gao, Yarong Wang, Fuqiang Zhang, Fei Sun, Xin Wang, Jiazhao Xie, Zhipeng Xu, Junjian Zhang, Haibo Xu, Yao Zhang, Jian‐Zhi Wang

**Affiliations:** ^1^ Key Laboratory of Ministry of Education for Neurological Disorders, Department of Pathophysiology, School of Basic Medicine, Tongji Medical College Huazhong University of Science and Technology Wuhan China; ^2^ Department of Neurology Zhongnan Hospital of Wuhan University, Wuhan University Wuhan China; ^3^ Department of Radiology Zhongnan Hospital of Wuhan University, Wuhan University Wuhan China; ^4^ Key Laboratory of Ministry of Education for Neurological Disorders, Department of Endocrine, Liyuan Hospital, Tongji Medical College Huazhong University of Science and Technology Wuhan China; ^5^ Co‐Innovation Center of Neuroregeneration Nantong University Nantong China; ^6^ Hubei Key Laboratory of Cognitive and Affective Disorders Jianghan University Wuhan China

**Keywords:** 9‐ING‐41, Alzheimer's disease, ApoE, glycogen synthase kinase‐3β, type 2 diabetes mellitus

## Abstract

**Background:**

The apolipoprotein E (ApoE) ε4 allele and type 2 diabetes mellitus (T2DM) are independent risk factors for Alzheimer's disease (AD), the most prevalent neurodegenerative disorder in the elderly. The T2DM patients carrying the ApoE ε4 allele exhibit heightened activation of platelet glycogen synthase kinase‐3β (GSK‐3β), a key downstream kinase in the insulin signaling pathway, along with more severe cognitive deficits. This observation suggests an intrinsic link between ApoE ε4, GSK‐3β, and cognitive dysfunction. However, the precise mechanisms by which ApoE ε4 influences GSK‐3β activity and exacerbates brain pathology and cognitive decline in T2DM patients remain poorly understood.

**Methods:**

To investigate these mechanisms, we developed T2DM mouse models by generating humanized ApoE ε3/ε3 and ε4/ε4 knock‐in mice. The mice were subjected to a high‐fat diet combined with multiple low‐dose intraperitoneal streptozotocin injections to induce T2DM. We then assessed GSK‐3β expression, AD‐like pathologies, and cognitive functions in these models.

**Results:**

We observed that GSK‐3β activity was significantly upregulated in ApoE4 mice, accompanied by disruption of the insulin signaling pathway. Notably, ApoE4‐T2DM mice exhibited exacerbated AD‐related pathologies, including increased accumulation of hyperphosphorylated tau, neuroinflammation, and synaptic loss. These changes were correlated with more severe cognitive impairments compared with ApoE3‐T2DM or ApoE4 mice. Furthermore, inhibition of GSK‐3β activity using the selective inhibitor 9‐ING‐41 effectively ameliorated both AD‐like pathologies and cognitive deficits in ApoE4‐T2DM mice.

**Conclusions:**

Our findings suggest that ApoE4 exacerbates AD pathogenesis by activating GSK‐3β. Furthermore, targeting GSK‐3β may offer a promising therapeutic strategy to halt the progression from T2DM to AD, providing new insights into potential interventions for patients at risk.

## Graphical abstract

ApoE4 exacerbates Alzheimer's‐like pathologies in T2DM by activating GSK‐3β and disrupting insulin signaling, leading to tau hyperphosphorylation, neuroinflammation, and cognitive decline. Inhibition of GSK‐3β using 9‐ING‐41 effectively ameliorates these pathologies, presenting a promising therapeutic approach to halt the progression from T2DM to AD.

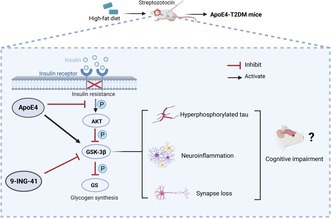

## 1 Introduction

Alzheimer's disease (AD) is the most prevalent neurodegenerative disorder and the leading cause of dementia in the elderly, imposing a considerable societal burden [1]. The hallmarks of AD include intracellular neurofibrillary tangles (NFTs) composed of aggregated hyperphosphorylated tau and extracellular senile plaques consisting of amyloid beta (Aβ), gliosis, neuronal loss, and synaptic dysfunction [2]. From a genetic standpoint, AD can be classified into familial Alzheimer's disease (FAD) and sporadic Alzheimer's disease (SAD). FAD usually presents before the age of 65 and is associated with mutations in the *Presenilin 1* (*PSEN1*), *amyloid precursor protein* (*APP*), and *Presenilin 2* (*PSEN2*) genes [3]. In contrast, over 95% of SAD cases typically manifest after the age of 65, with an etiology that remains unclear and is likely influenced by multiple complex factors [4].

SAD results from a multifactorial interaction between genetic predispositions and environmental influences. The ε4 isoform of apolipoprotein E (ApoE4) remains a major genetic risk factor for SAD [5]. ApoE is a lipid‐binding protein involved in the transport and metabolism of cholesterol and lipids and exists in humans as three major isoforms: ApoE2, ApoE3, and ApoE4 [6]. Carrying one copy of ApoE ε4 increases the risk of developing AD threefold, while individuals with two copies of ApoE ε4 face a 90% lifetime probability of developing the condition [7]. Conversely, ApoE2 is associated with a lower risk of AD compared with the ApoE3, which is considered the wild‐type variant and is the most common among the three isoforms [8, 9]. Environmental factors, particularly type 2 diabetes mellitus (T2DM), significantly increase the risk of developing AD. Individuals with diabetes have a 1.5–3 times higher likelihood of experiencing cognitive impairments compared with those without diabetes [10, 11]. T2DM accounts for approximately 90%–95% of all diabetes cases globally, is characterized by chronic hyperglycemia resulting from insulin resistance or insufficient insulin secretion [12]. T2DM shares several pathophysiological features with AD, including impaired glucose metabolism, insulin resistance, inflammation, amyloid protein accumulation, and cognitive decline [12, 13, 14]. Owing to these similarities, some researchers have come to refer to AD as “brain diabetes” or “type 3 diabetes” [12, 15].

Although the exact mechanisms by which T2DM increases the risk of AD are not fully understood, evidence suggests cognitive impairments in T2DM patients linked to disrupted insulin signaling [16, 17]. Glycogen synthase kinase‐3β (GSK‐3β) plays a critical role in the insulin signaling pathway and contributes to the pathogenesis of both AD and T2DM [18, 19, 20]. Under physiological conditions, insulin binds to its receptor, initiating downstream signaling pathways that activate protein kinase B (PKB/AKT). Activated AKT then phosphorylates GSK‐3β at the Ser9 residue, leading to the inactivation of GSK‐3β [21, 22, 23]. This process promotes glycogen synthesis, thereby regulating blood glucose levels [22]. However, in T2DM, the insulin signaling pathway is compromised, resulting in the activation of GSK‐3β. This activation not only enhances tau phosphorylation and promotes neuroinflammation but also disrupts glucose homeostasis by affecting glycogen synthesis [24, 25]. The presence of ApoE ε4 gene and upregulation of platelet GSK‐3β activity are respectively correlated with the occurrence of mild cognitive impairment in T2DM patients [26], and individuals carrying the ApoE ε4 allele exhibited elevated GSK‐3β activity in platelets and more severe cognitive impairment compared with those with the ApoE ε3 allele within a cohort of T2DM patients [27, 28]. These studies suggest an intrinsic link among ApoE4, GSK‐3β and cognitive deficits in T2DM patients. However, whether and how ApoE4 exacerbates cognitive deficits through GSK‐3β in T2DM conditions are not known.

In the present study, we established T2DM mouse models by using a high‐fat diet combined with intraperitoneal streptozotocin injections in humanized ApoE3 and ApoE4 knock‐in mice [29, 30]. We found that expressing ApoE4 upregulated GSK‐3β in the hippocampus and exacerbated AD‐like pathologies and cognitive impairment in T2DM mice. Furthermore, the application of GSK‐3β inhibitor, 9‐ING‐41, significantly alleviated the AD‐like pathologies and cognitive deficits in ApoE4‐T2DM mice.

## 2 Materials and Methods

### 2.1 Animals

All mice were housed at 23°C–25°C under a 12‐h light/dark cycle. Food and water were provided ad libitum throughout the experiment. All animal experiments were approved by the Institutional Animal Care and Use Committee of Tongji Medical College, Huazhong University of Science and Technology. The humanized ApoEε3/ε3 knock‐in mice (ApoE3 KI mice, RRID: 029018) and ApoEε4/ε4 knock‐in mice (ApoE4 KI mice, RRID: 027894) were generated by the Jackson Laboratory. In the ApoE4 KI mice, exons 2, 3, and the majority of exon 4 of the endogenous ApoE gene were replaced by the human ApoE gene sequence. To generate ApoE3 KI mice, CRISPR/Cas9 genome editing was employed to introduce a C‐to‐T nucleotide substitution into the human APOE sequence of the ApoE4 KI allele, resulting in the R130C mutation. The animals were distributed into various experimental groups with balanced littermates and gender, ensuring each group contained an equivalent number of male and female siblings.

### 2.2 Antibodies and Reagents

Antibodies and reagents used in this study are listed as Table S1.

### 2.3 HFD‐ and STZ‐Induced T2DM Mice

To establish a type 2 diabetes mellitus (T2DM) model in 10‐week‐old mice, a 60% high‐fat diet (HFD, D12492; Research Diets) was fed for 8 weeks. Following this dietary intervention, streptozotocin (STZ, 30 mg/kg, 572201; Sigma‐Aldrich) was dissolved in citrate buffer (pH 4.35) and administered via intraperitoneal injection over five consecutive days. Control mice received an equivalent volume of citrate–phosphate buffer. Prior to each injection, all mice were fasted for 6–8 h, with free access to water maintained. Individual body weights were recorded to accurately determine the dose of STZ. Given the instability of STZ, the solution was prepared immediately before use, kept away from light, and administered within 30 min to ensure efficacy. The successful induction of the T2DM model was confirmed by fasting blood glucose levels exceeding 11.1 mmol/L.

### 2.4 9‐ING‐41 Injection

To prepare a solution at a concentration of 4 mg/mL, mix 10% DMSO with 20% PEG400 to dissolve the GSK‐3β inhibitor 9‐ING‐41 (Elraglusib, HY‐113914; MCE). Due to the limited solubility of 9‐ING‐41, ultrasonic assistance may be utilized to enhance the dissolution process. The solution should be freshly prepared for each use. Administer the drug via intraperitoneal injection at a dosage of 20 mg/kg in 19‐week‐old ApoE4‐T2DM mice, ensuring accurate calculation of the injection volume for each mouse. Injections should be conducted three times a week at the same time, continuing for a total duration of 4 weeks.

### 2.5 Liver Damage and Cholesterol Metabolism Analysis

Following the collection of blood from the mice via orbital puncture, transfer the samples into appropriately labeled Eppendorf tubes. Allow the samples to stand overnight at 4°C. Subsequently, centrifuge the samples at 3000 rpm for 15 min at 4°C. Carefully transfer the supernatant into new, labeled Eppendorf tubes. Transport the samples on dry ice to Servicebio Technology Co. Ltd. (Wuhan) for analysis. An automatic biochemical analyzer (Chemray 240) from Leadbio Life Science Technology Co. Ltd. (Shenzhen) will be employed for the measurements.

### 2.6 Western Blotting

The skulls of the mice were carefully removed, and the hippocampal tissue was dissected on ice. The samples were homogenized in RIPA lysis buffer containing PMSF (Millipore) and a protease inhibitor cocktail (Sigma) at a ratio of 10 μL per mg of tissue. Ultrasonic disruption was performed on ice for 10–15 s, followed by centrifugation at 12,000 rpm for 15 min at 4°C. The supernatant was collected, and the protein concentration was assessed using the BCA assay kit (Thermo Fisher). Proteins were then separated by SDS‐PAGE (10%) for approximately 1.5 h and subsequently transferred to nitrocellulose membranes (Merck Millipore; 0.45 μm) for 1 h. The membranes were blocked with 5% bovine serum albumin (BSA) for 1 h. Following this, the membranes were incubated sequentially with primary and secondary antibodies. Protein bands were visualized using an ECL Imaging System (610007‐8Q; Clinx Science Instruments Co. Ltd.) and quantified with ImageJ software.

### 2.7 Immunostaining and Quantification

Mice were anesthetized with 2% isoflurane 1 day post‐behavioral testing and perfused intracardially with 0.9% NaCl followed by 4% paraformaldehyde (PFA) in 0.1 M phosphate buffer (pH 7.4). Brains were removed and cryoprotected in 25% and 30% sucrose for 2 days, then sectioned into 30 μm slices using a cryostat (Leica CM1900). For immunohistochemistry, free‐floating sections were treated with 3% H_2_O_2_ in methanol for 30 min to block endogenous peroxidase activity, followed by blocking with 5% BSA for 30 min at room temperature. Sections were incubated with primary antibodies overnight at 4°C, then dehydrated through a graded ethanol series (75%, 95%, 100%) and cleared in xylene (two 10‐min rounds). Slices were mounted with neutral balsam, dried in the dark, and immunoreactivity visualized using a DAB staining kit (ZSGB‐BIO). Images were captured with an automatic slide scanner (SV120; Olympus). For immunofluorescence, sections were blocked in 5% BSA with 0.1% Triton X‐100 for 30 min at room temperature, incubated with primary antibodies overnight at 4°C, washed in PBS (three 5‐min washes), and incubated with secondary antibodies at 37°C for 1 h. Nuclei were counterstained with DAPI. Images were obtained using an automatic slide scanner (SV120; Olympus) and a confocal microscope (Zeiss Carl LSM 800). Quantitative analysis was conducted using ImageJ to assess the staining area and the number of GFAP‐ and Iba1‐positive cells. One slice per mouse was analyzed, with 6–7 mice per group.

### 2.8 Thioflavin S Staining

Brain sections were rinsed with phosphate‐buffered saline (PBS) for 15 min and subsequently stained with 0.3% Thioflavin S (#1326‐12‐1; Sigma) in 50% ethanol at room temperature for 10 min. The staining duration was adjusted according to the observed intensity under the microscope. After staining, the sections were decolorized in 50% ethanol for 3–5 min, washed with PBS, and mounted using a coverslipping medium containing DAPI.

### 2.9 Nissl Staining

After mounting the brain sections on adhesive slides, they were stained using Nissl stain for 10 min. The sections were subsequently decolorized in 75% ethanol for approximately 5 min, followed by sequential treatments with 80%, 90%, and 100% ethanol for 2 min each. The staining and decolorization durations were adjusted based on the observed staining intensity under the microscope. Following dehydration, the sections were cleared in xylene for 1 h and mounted using neutral balsam.

### 2.10 Enzyme‐Linked Immunosorbent Assay (ELISA)

For mouse serum, orbital blood was collected and incubated at 4°C overnight. The sample was then centrifuged at 3000 rpm for 15 min, and the supernatant was analyzed immediately. For mouse cortical tissue, nine volumes of physiological saline were added to the tissue (1 mg of tissue to 9 μL of saline). The mixture was homogenized on ice to produce a 10% homogenate and then centrifuged at 3000 rpm for 10 min. The supernatant was analyzed according to the instructions of the reagent kit, and Aβ40 and Aβ42 levels were detected using commercial ELISA kits (E‐EL‐H0542c 90 and E‐EL‐H0543c; Elabscience).

### 2.11 Golgi Staining and Spine Analyses

After the mouse was anesthetized with 2% isoflurane, the brain tissue was carefully extracted by removing the skull. The tissue was then immersed in Golgi staining solution, which was prepared by mixing 5% potassium dichromate, 5% silver nitrate, and an equal volume of 5% potassium dichromate solution in a 1:1:2 ratio for 2 weeks in the dark. Following the staining process, the tissue was transferred to a preservation solution containing 300 g of sucrose, 500 mL of phosphate buffer (PB), 200 mL of ethanol, and 4 g of polyvinylpyrrolidone for 2 days, also in the dark. Sections of 100 μm thickness were cut while the brain was immersed in PB.

The sections underwent a series of washes: twice in distilled water (3 min each), followed by a rinse in 50% ethanol for 5 min, and then treatment with a 66% ammonia solution for approximately 10 min. They were rinsed again twice in distilled water (5 min each), incubated in 5% sodium thiosulfate for 10 min in the dark, and subsequently washed twice more in distilled water (5 min each). The sections were mounted on slides with 2% gelatin, dehydrated through a graded series of ethanol (75%, 85%, 90%, 100%) for 2 min each, cleared in xylene for 20 min, and finally sealed with neutral gum. Subsequently, these sections were imaged using a light microscope (Nikon, Japan) for further analysis. Spine densities were quantified as the number of spines per 10 mm of dendrite length, measured using ImageJ 2.0 software.

### 2.12 Open Field Test

During the experiment, the mouse was placed in a square enclosure measuring 60 × 60 × 50 cm^3^. The floor of the enclosure was evenly divided into 16 squares, including a central zone (comprising the central four squares) and 12 peripheral zones. The mouse was introduced into the enclosure with its back facing the experimenter and was allowed to explore freely for 5 min. The environment was kept quiet throughout the test, and a video tracking system (Taimeng Software Co. Ltd., Chengdu, China) was used to record and analyze the mouse's behavior.

### 2.13 Novel Object Recognition Test

The experiment lasted 2 days. On the first day, two identical objects, A and B, were placed in opposite corners of the enclosure, and the mouse was allowed to explore the objects for 5 min. On the following day, object B was replaced with a novel object C, and the mouse was also given 5 min to explore the objects. The video tracking system (Anymaze Technology SA, Stoelting Co., IL) identified the mouse's exploration of the objects based on the proximity of the mouse's head to each object. The exploration times for objects A and C were recorded as TA and TC, respectively, and the discrimination index was calculated as (TC − TA)/(TC + TA). Throughout the experiment, the environment was kept quiet, and handling of the mouse was gentle. Before testing each new mouse, the enclosure was cleaned of feces and disinfected with 75% ethanol.

### 2.14 Object Place Recognition Test

The experiment was conducted over 2 days. On Day 1, two identical objects, A and B, were placed in opposite corners of the enclosure, and the mouse was allowed to explore the objects for 5 min. On the following day, object B was relocated to a different corner of the enclosure while object A remained in the same corner. The mouse was also given 5 min to explore the objects. The video tracking system (Anymaze Technology SA, Stoelting Co., IL) recorded the mouse's exploration of the objects based on the proximity of the mouse's head to each object. The exploration times for objects A and B were recorded as TA and TB respectively. On Day 2, the discrimination index was calculated as (TB − TA)/(TB + TA). Throughout the experiment, the environment was kept quiet, and handling of the mouse was gentle. Before testing each new mouse, the enclosure was cleaned of feces and disinfected with 75% ethanol.

### 2.15 Morris Water Maze Test

The water maze (a circular pool, 1.2 m in diameter and 0.6 m in height) was divided into four quadrants, with a platform placed 2 cm below the water surface in one quadrant. The pool's walls were marked with brightly colored signs, and a camera system (Taimeng, China) was used to track the mice's movements and speed. Before the experiment, mice were acclimated in the experimental room for at least 6 h, and heaters and dry bedding were prepared. The learning phase lasted 5 days, with three trials per day and at least a 30‐min interval between trials in each quadrant. Mice were gently placed in the water to find the platform, with a maximum swim time of 60 s per trial. If a mouse found the platform, its time was recorded, and it was allowed to remain on the platform for 15 s. If the platform was not located within 60 s, the mouse was guided to it and allowed to stay for 15 s. On Day 6, spatial memory was assessed by removing the platform and placing the mice in the quadrant opposite to the platform's original location. Mice were given 60 s to search for the platform, and the number of crossings over the former platform location was recorded, along with the percentage of distance moved in the target quadrant.

### 2.16 Statistical Analysis

Data analysis and plotting were performed using GraphPad Prism 8 (La Jolla, CA). The Shapiro–Wilk test was used to evaluate normality, and non‐parametric tests were employed when required. Unpaired *t* test with Welch's correction was used for comparisons between two groups. For comparisons involving three or more groups, either one‐way ANOVA and Bonferroni's multiple comparison test or two‐way ANOVA and Bonferroni's post hoc test were applied for multiple comparisons. All experiments and analysis were performed in a blind manner, and experiments were replicated at least three times to ensure sufficient statistical power. Data are expressed as mean ± SEM or min‐median‐max unless otherwise specified. The probability (*p*) value < 0.05 was considered statistically significant.

## 3 Results

### 3.1 Generation and Validation of Humanized ApoE3/4‐T2DM Mouse Models

**FIGURE 1:**
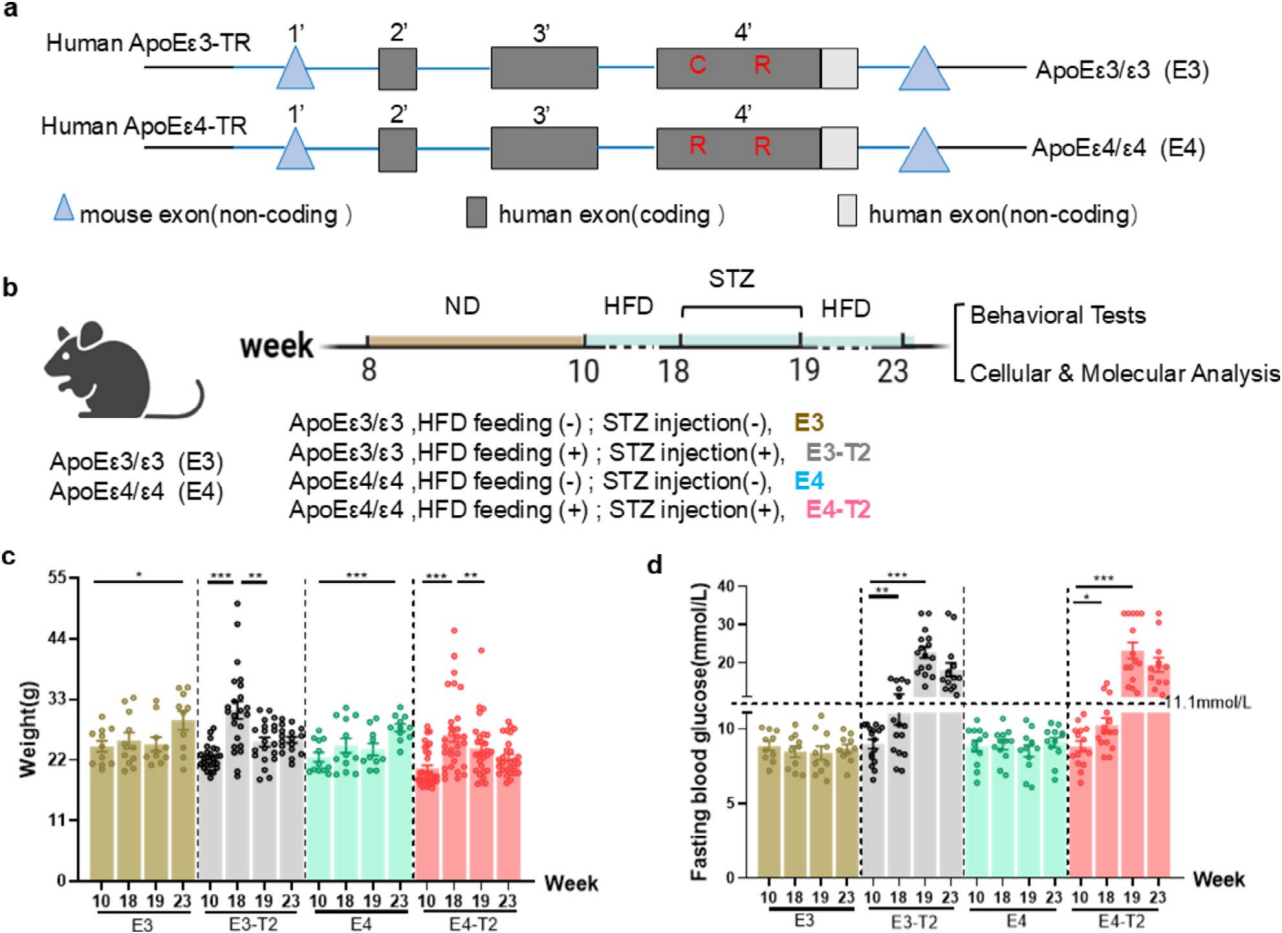
Generation of T2DM mouse models in humanized ApoE3 and ApoE4 mice. (a) Strategy for generating humanized ApoE3 and ApoE4 knock‐in mice. Exons 2, 3 and most of exon 4 of the mouse ApoE gene were replaced by human ApoE gene sequence including exons 2, 3 and 4. (b) The experiment protocol of T2DM mice models. (c) The weight and (d) fasting blood glucose during the modeling period. E3, *n* = 10. E3‐T2, *n* = 26. E4, *n* = 12. E4‐T2, *n* = 32. Unpaired *t*‐test with Welch's correction, *p < 0.05, **p < 0.01, ***p < 0.001, data presented as mean ± SEM.

Rodents possess a single ApoE genotype that closely resembles human ApoE4 [31, 32]. To investigate the potential underlying mechanisms and the related preventive strategies for ApoE4‐exacerbated cognitive impairment in T2DM, we developed T2DM mouse models in humanized ApoE3 and ApoE4 knock‐in mice (Figure 1a). The mice carrying either the ApoE3 or ApoE4 genotype were randomly assigned to two groups: a normal diet group and a T2DM model group with an equal ratio of male to female mice maintained in each group. The T2DM model group was fed a high‐fat diet for 8 weeks, followed by intraperitoneal injections of streptozotocin (30 mg/kg/day for five consecutive days) (Figure 1b). During the model induction, body weight and blood glucose levels were dynamically monitored (Figure 1c,d). A fasting blood glucose level exceeding 11.1 mmol/L or a random blood glucose level greater than 16.7 mmol/L was considered indicative of successful establishment of the T2DM model [33, 34]. Furthermore, we performed weekly random blood glucose measurements for 4 weeks after STZ injection in ApoE3‐T2DM and ApoE4‐T2DM mice that continued to be fed a high‐fat diet to rule out non‐specific toxicity to brain tissue (Figure S1a).

T2DM mice exhibited symptoms resembling those of clinical diabetes patients, including hyperglycemia and body weight loss (Figure 1c,d). Additionally, T2DM mice displayed liver enlargement, a gray‐white appearance, a firm texture, and an oily surface film (Figure S1b). Compared with E3‐T2 or E4 mice, E4‐T2 mice exhibited a significantly increased liver index (Figure S1c), while serum alanine aminotransferase (ALT) and aspartate aminotransferase (AST) levels remained unchanged (Figure S1d,e). Furthermore, T2DM mice showed elevated serum levels of triglycerides (TG), cholesterol (CHO), and low‐density lipoprotein (LDL) compared with the normal diet group (Figure S1f–i). We measured the serum glucose (GLU) and glycated serum protein (GSP) levels in the mice, noting that while GLU reflects immediate glycemic status, GSP indicates average blood glucose levels over the past few weeks. Both GLU and GSP levels were significantly higher in T2DM mice compared to non‐T2DM mice (Figure S1j,k). These results confirm that both ApoE3‐T2DM and ApoE4‐T2DM mice showed elevated blood glucose, and an exacerbated hepatic lipid accumulation was seen in ApoE4‐T2DM mice.

### 3.2 ApoE4 Exacerbated Aggregation of Phosphorylated Tau With Limited Effect on Aβ Precipitation in T2DM Mice

**FIGURE 2:**
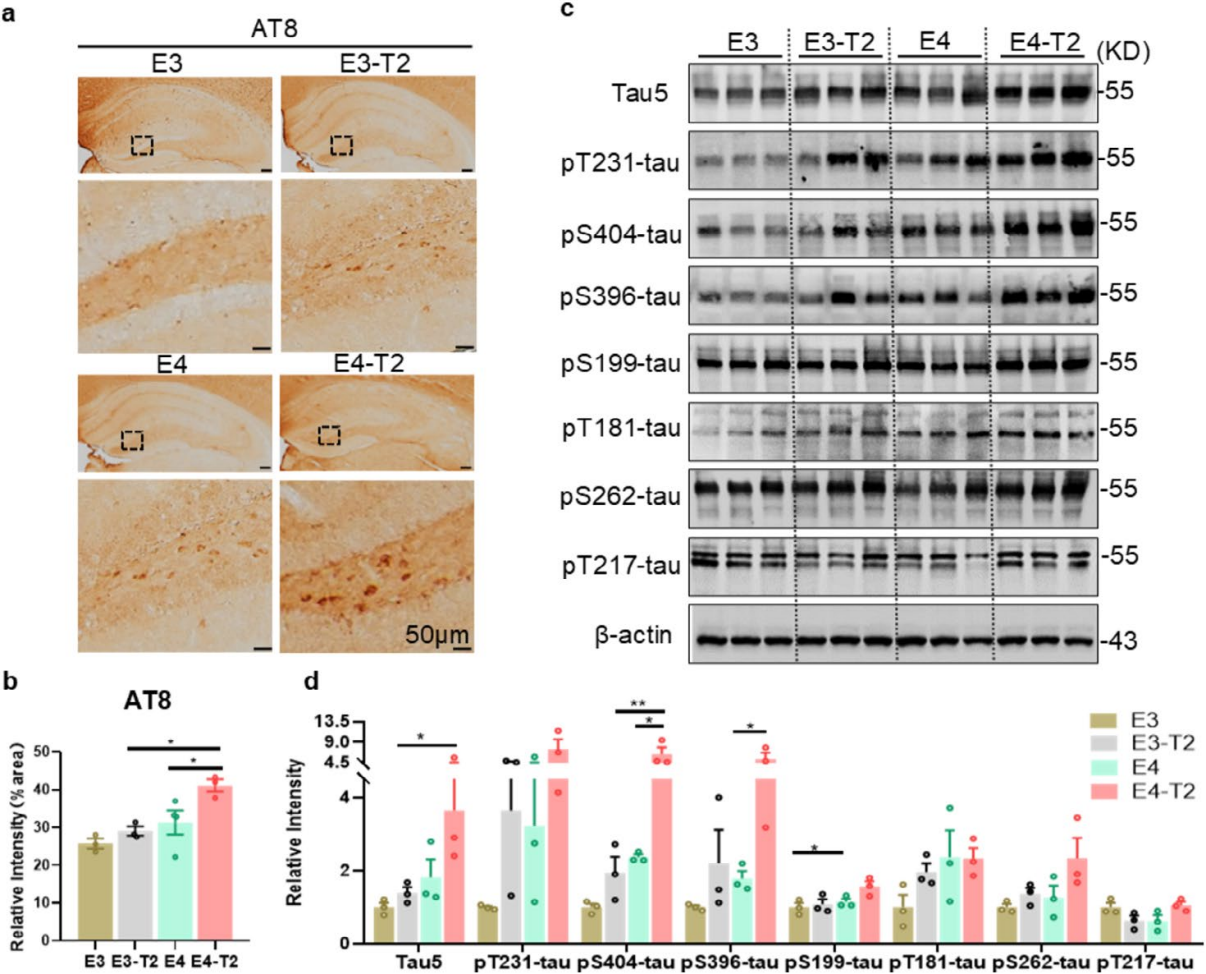
ApoE4 exacerbated phosphorylated tau aggregation in the hippocampus of T2DM mice. (a) Representative immunohistochemical staining images of AT8 (Ser202/Thr205) in mice and (b) the percentage of AT8 positively stained area in the DG of the hippocampus. Scale bar = 50 μm, three mice per group, two‐way ANOVA followed by Bonferroni's tests. (c) The levels of total tau protein and multiple phosphorylation sites of tau protein in the hippocampus of mice and (d) quantitative analysis. Three mice per group, two‐way ANOVA followed by Bonferroni's tests, 6‐month‐old mice, *p < 0.05, **p < 0.01, data presented as mean ± SEM.

GSK‐3β is a critical enzyme involved in the phosphorylation of tau protein [35], and the aggregation of hyperphosphorylated tau is a hallmark of AD [36]. To determine whether ApoE4 affects the phosphorylation of tau protein in T2DM mice, we assessed the levels of total tau (detected by tau5 antibody) and tau phosphorylation at multiple AD‐related epitopes in the hippocampus of mice. Immunohistochemical staining revealed that the levels of phosphorylated tau at pS202/pT205 sites (detected by AT8 antibody) were significantly increased in 6‐month‐old E4‐T2 mice compared with E3‐T2 and E4 mice (Figure 2a,b). Additionally, Western blotting analysis indicated that both total tau and phosphorylated tau levels (including the pS404, pS396, and pS199 epitopes) were significantly elevated in the hippocampus of E4‐T2 mice compared with E3‐T2 mice. Furthermore, ApoE4 enhanced tau phosphorylation at the pS404, pS396, and pS199 epitopes in non‐diabetic mice (Figure 2c,d). These results suggest that both ApoE4 and T2DM contribute to increased tau phosphorylation, with the most pronounced AD‐like tau pathologies observed in ApoE4‐T2DM mice.

Abnormal aggregation of β‐amyloid (Aβ), forming senile plaques, is a hallmark of AD [37]. Studies have indicated that ApoE plays a critical role in regulating the metabolism and clearance of Aβ [38]. Specifically, the ApoE4 allele may increase the risk of AD by accelerating the aggregation and deposition of Aβ in the brain [37, 39]. To investigate the potential influence of ApoE4 on Aβ pathology in T2DM mice, we performed Thioflavin S staining on 6‐month‐old mice. No positively stained plaques were observed in these mice, unlike the age‐matched 5xFAD mice, which express human APP and PSEN1 transgenes harboring five AD‐associated mutations and display significant Aβ plaque deposition in the hippocampus, especially in the DG region (Figure S2a). Then, we quantified the Aβ42/Aβ40 ratio in both brain and serum using ELISA. We found that E4‐T2 mice exhibited an increased Aβ42/Aβ40 ratio in the cortex compared with E3‐T2 mice (Figure S2b). Additionally, no significant difference but an increasing trend of Aβ42/Aβ40 ratio was observed in the serum of E4‐T2 mice compared with E3‐T2 mice (Figure S2c). These results suggest that the influence of ApoE4 and T2DM on the brain Aβ deposition is limited.

### 3.3 ApoE4 Exacerbated Neuroinflammation, Synaptic Damages, and Cognitive Deficits in T2DM Mice

**FIGURE 3:**
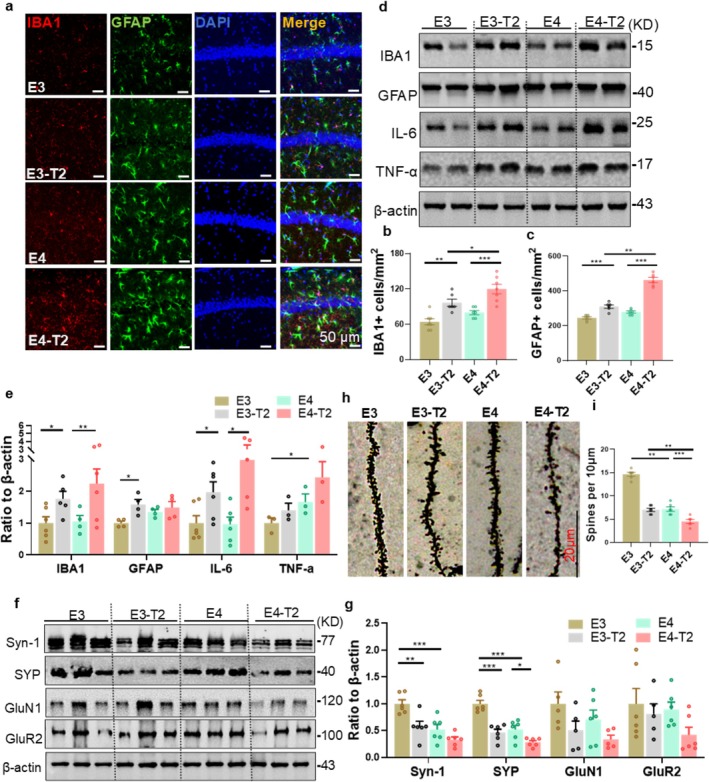
ApoE4 promoted gliosis, neuroinflammation, and synaptic deficits in the hippocampus of T2DM mice. (a) Representative immunofluorescence staining of microglia and astrocytes. (b) Microglial cell count in the hippocampal CA1 region (1 mm^2^) and (c) astrocyte count. Scale bar = 50 μm, six mice per group, two‐way ANOVA followed by Bonferroni's tests. (d) Expression levels of neuroglial cells in mouse hippocampal tissue and associated inflammatory factor protein expression with (e) quantification results. Three to six mice per group, two‐way ANOVA followed by Bonferroni's tests. (f) Expression levels of synaptic‐related proteins in the hippocampal tissue of mice and (g) quantitative results. Six mice per group, two‐way ANOVA followed by Bonferroni's tests. GluN1, NMDA receptor subunit 1; GluR2, glutamate receptor 2; Syn‐1, synapsin‐1; SYP, synaptophysin. (h) Golgi staining results in the DG of the mouse hippocampus and (i) comparison of the number of dendritic spines with a length of 10 μm. Scale bar = 20 μm, six mice per group, two‐way ANOVA followed by Bonferroni's tests. 6‐month‐old mice, *p < 0.05, **p < 0.01, ***p < 0.001, data presented as mean ± SEM.

Accumulating evidence has suggested that gliosis and neuroinflammation play pivotal roles in the pathology of AD [40]. Immunofluorescence staining showed that ApoE4 significantly increased the number of GFAP‐labeled astrocytes and IBA1‐labeled microglia in hippocampal CA1 of T2DM mice (Figure 3a–c). Furthermore, the number of astrocytes and microglia was increased in T2DM mice with different humanized ApoE genotypes (Figure 3a–c). Activated microglia play a central role in the progression of neurodegenerative diseases, including AD, by modulating inflammatory responses through the release of various mediators, which subsequently affect the overall function and structure of the nervous system [41]. These inflammatory mediators include tumor necrosis factor‐alpha (TNF‐α), interleukin‐6 (IL‐6), and nitric oxide (NO) [42]. Western blotting analysis showed that the IL‐6 expression level was increased in the hippocampus of T2DM mice with different humanized ApoE genotypes (Figure 3d,e). Additionally, TNF‐α expression was increased in E4 mice compared with E3 mice (Figure 3d,e). These observations suggest that T2DM promotes hippocampal glia‐associated expression of inflammatory factors and ApoE4 exacerbates the effects.

The aggregation of phosphorylated tau and activation of glial cells are closely linked to synaptic impairment. Severe synaptic deficits may lead to neuronal death [43]. To evaluate the impact of ApoE4 and T2DM on synaptic integrity, we measured the levels of synapse‐related proteins and spine density in the hippocampus of mice. Western blotting analysis showed that both ApoE4 and T2DM significantly reduced the levels of presynaptic synapsin‐1 (Syn‐1) and synaptophysin (SYP). Additionally, ApoE4 decreased the levels of postsynaptic NMDA receptor subunit 1 (GluN1) and glutamate receptor 2 (GluR2), although these changes were not statistically significant (Figure 3f,g). Golgi staining showed that spine density in the hippocampal DG was significantly decreased in the E4‐T2 and E4 groups compared with the E3‐T2 and E3 groups, respectively (Figure 3h,i). These findings suggest that ApoE4 exacerbates synaptic deficits in both T2DM and non‐T2DM mice, and T2DM also contributes to synaptic damage. Additionally, neither ApoE4 nor T2DM led to decreased neuron numbers in the hippocampal CA1 subset during the observed period, as assessed by Nissl staining (Figure S3a,b). Collectively, these findings demonstrate that T2DM and ApoE4 exacerbate synaptic deficits without inducing neuronal loss in mice.

**FIGURE 4:**
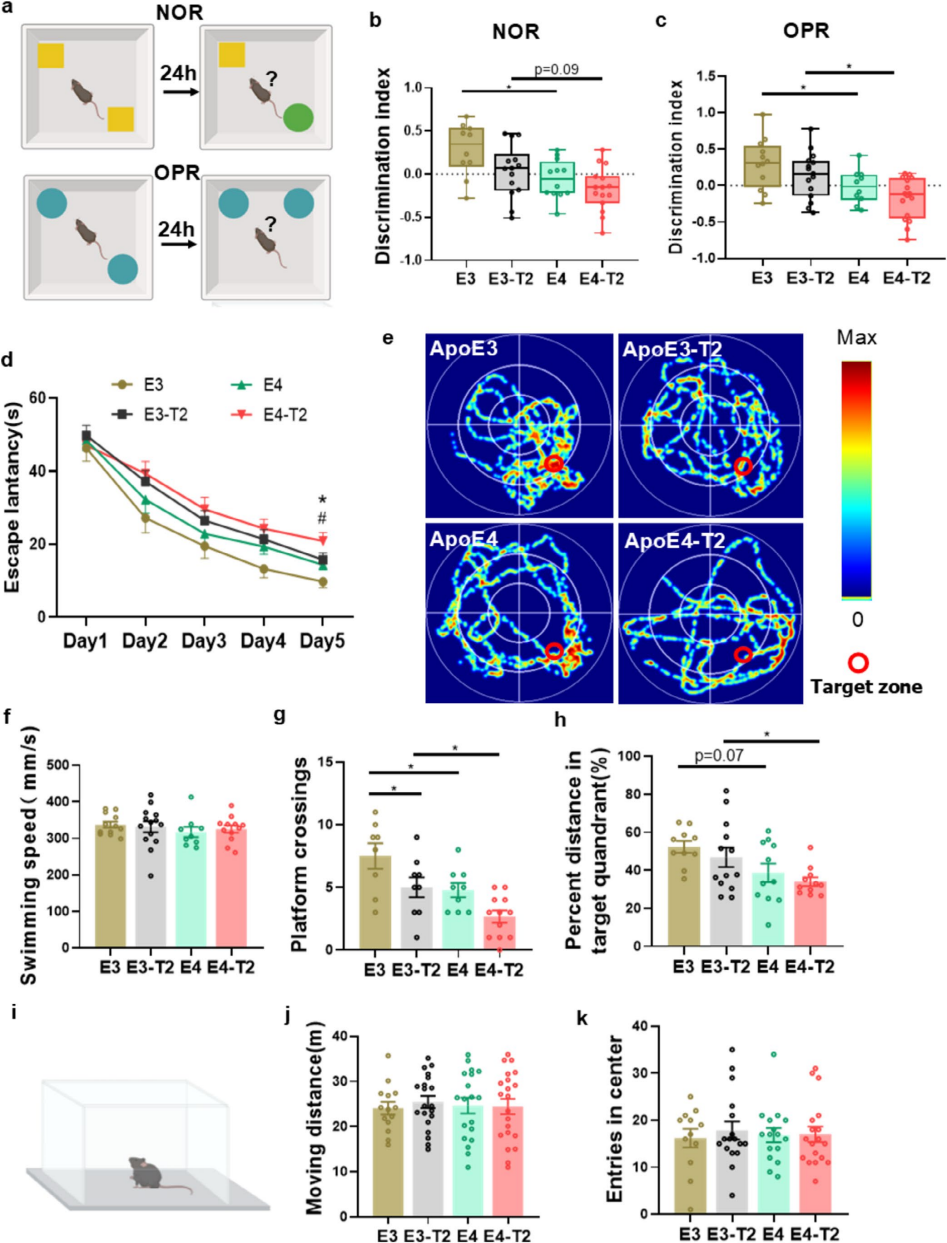
ApoE4 exacerbated learning and memory impairments in T2DM mice. (a) The behavioral paradigms of NOR and OPR experiment. (b, c) Statistical results of the NOR and OPR discrimination index for each group of mice. 10–14 mice per group, two‐way ANOVA followed by Bonferroni's tests. (d) Latency during the learning phase of the MWM experiment. *p < 0.05, E3‐T2 versus E4‐T2. ^#^ *p* < 0.05, E4 versus E4‐T2. (e) Representative heatmaps of movement trajectories during the detection phase of the MWM experiment. (f) Average swimming speed during the detection phase of the MWM experiment. (g) Platform crossing frequency during the MWM experiment for each group of mice. (h) Target quadrant movement distance during the MWM experiment. 10–13 mice per group, two‐way ANOVA followed by Bonferroni's tests. (i) Open field test (OFT) behavioral paradigm. (j) Total distance traveled in the OFT experiment and (k) number of entries into the center zone during the OFT experiment. 14–20 mice per group, two‐way ANOVA followed by Bonferroni's tests. Six‐month‐old mice, *p < 0.05, data presented as mean ± SEM.

To investigate the effects of ApoE4 and T2DM on learning and memory, we conducted a series of behavioral tests to assess cognitive function in mice. In the novel object recognition (NOR) test, E4‐T2 mice showed a decreased discrimination index for the novel object compared with E3‐T2 mice. Similarly, E4 mice exhibited a decline in the discrimination index compared with E3 mice (Figure 4a,b). In the object place recognition (OPR) test, both E4‐T2 and E4 mice displayed poorer performance in discriminating objects moved to a new location than E3‐T2 and E3 mice (Figure 4a,c). During the learning trial of the Morris water maze (MWM) test, E3‐T2 mice showed longer latency on day 4 than E3 mice, while E4‐T2 mice exhibited prolonged latency on day 5 compared with E3‐T2 or E4 mice (Figure 4d). During the probe test on day 6, E4‐T2 mice showed fewer platform crossings (Figure 4e,g) and a lower percentage of distance traveled in the target quadrant (Figure 4h) compared with E3‐T2 mice. No significant differences were observed among the four groups in swimming speed (Figure 4f). Importantly, correlation analysis revealed a significant negative relationship between tau phosphorylation at multiple epitopes (pT231, pS404, pS396, and pS199) in the hippocampus and the number of platform crossings, demonstrating that increased tau phosphorylation is associated with worse spatial memory performance (Figure S4a–d). These data suggest that both ApoE4 and T2DM impair spatial learning and memory in mice. In the open field test, no significant differences were observed in total distance traveled or center area entries among the four groups of mice (Figure 4i–k), suggesting that neither ApoE4 nor T2DM significantly affects anxiety‐like behavior. Collectively, these results demonstrate a synergistic effect of ApoE4 and T2DM on cognitive impairment in mice, and ApoE4‐T2DM mice show the most severe cognitive deficits.

### 3.4 ApoE4 Exacerbated Insulin Signaling Dysfunction and Upregulated GSK‐3β in the Hippocampus of T2DM Mice

**FIGURE 5:**
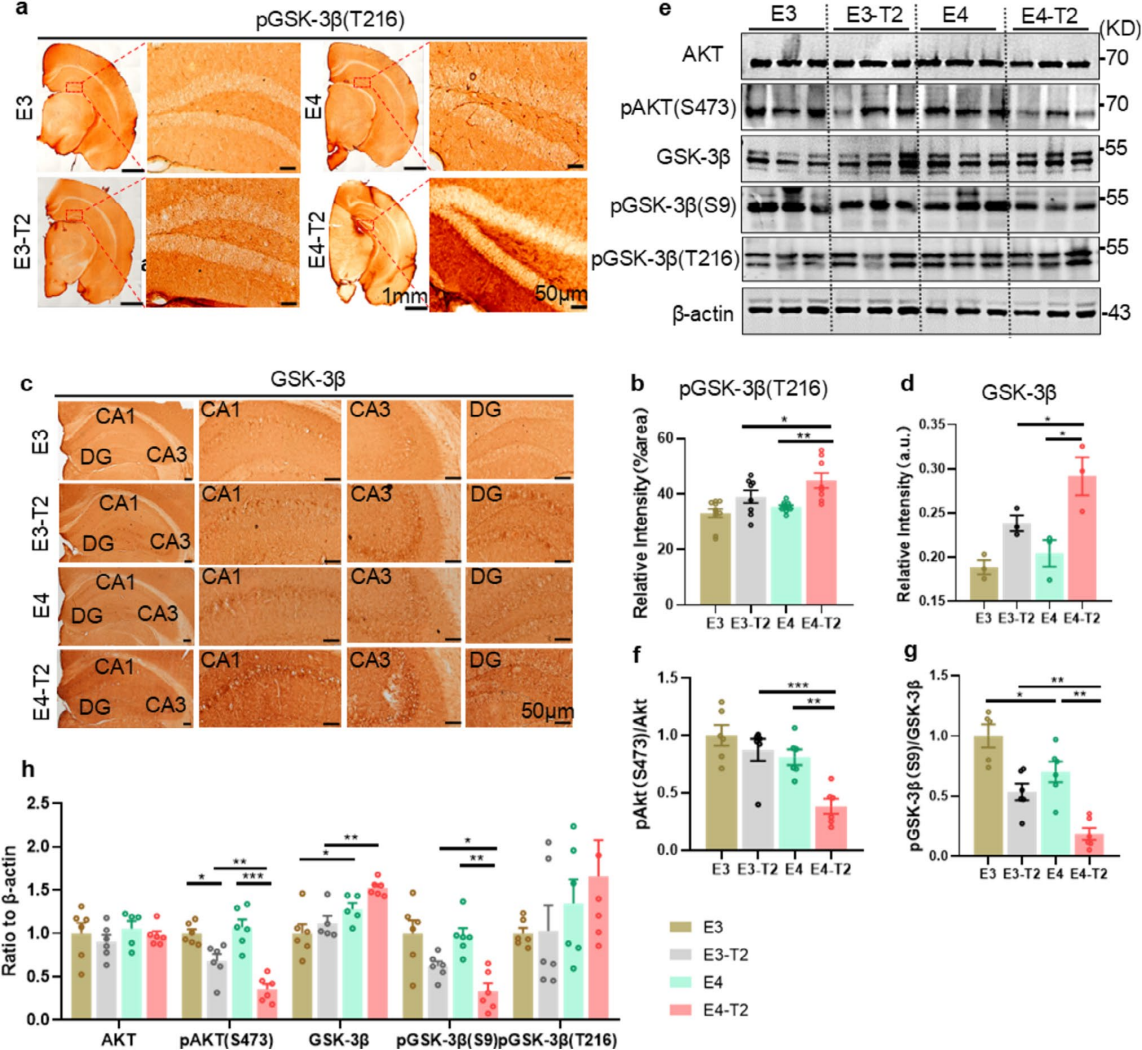
ApoE4 exacerbated insulin signaling impairment and upregulated GSK‐3β in the hippocampus of T2DM mice. (a) Representative immunohistochemical staining of pGSK‐3β (T216) and (b) percentage of positively stained area in the dentate gyrus (DG) of the hippocampus. Scale bar = 50 μm, 8–10 mice per group, two‐way ANOVA followed by Bonferroni's tests. (c) Immunohistochemical staining of GSK‐3β in mice and (d) quantification of results. Scale bar =50 μm. Three mice per group, two‐way ANOVA followed by Bonferroni's tests. (e) Changes in insulin signaling pathway‐related proteins in mouse hippocampal tissue and (f–h) quantification analysis. Six mice per group, two‐way ANOVA followed by Bonferroni's tests. Six‐month‐old mice, *p < 0.05, **p < 0.01, ***p < 0.001, data presented as mean ± SEM.

Our previous studies have shown that T2DM patients carrying the ApoE4 allele exhibit increased activity of GSK‐3β in platelets and more severe cognitive impairment [27, 28]. To investigate whether ApoE4 influences cerebral GSK‐3β expression and the insulin signaling pathway in T2DM mice, we first assessed the levels of total GSK‐3β (tGSK‐3β) and phosphorylated GSK‐3β at Tyr216 (pGSK‐3β‐Tyr216, active form) in 6‐month‐old mice (Figure 5a,b). Immunohistochemical analysis showed significantly increased expression of tGSK‐3β and the phosphorylated pGSK‐3β‐Tyr216 in the hippocampal dentate gyrus (DG) of E4‐T2 mice compared with E3‐T2 mice (Figure 5a–d). This finding was further validated by Western blotting analysis, which showed consistent upregulation of tGSK‐3β and pGSK‐3β‐Tyr216 in the hippocampus of E4‐T2 mice (Figure 5e,h). These results suggest that ApoE4 enhances GSK‐3β activation in T2DM mice.

GSK‐3β is an essential component of the insulin signaling pathway. Under physiological conditions, upstream PI3K‐AKT signaling pathways phosphorylate GSK‐3β at Ser9, leading to its inactivation [21, 22, 23]. This modification promotes glycogen synthesis and reduces blood glucose levels [22]. Studies have indicated that ApoE4 interacts with insulin receptors, thereby impairing cerebral insulin signaling [44]. Therefore, we examined the expression of insulin‐related signaling molecules in the hippocampus of 6‐month‐old mice A significantly reduced phosphorylated AKT at Ser473 (pAKT‐Ser473) with phosphorylated GSK‐3β at Ser9 (pGSK‐3β‐Ser9) was detected in the hippocampus of E4‐T2 mice compared with E3‐T2 and E4 mice (Figure 5e–h). Additionally, a significantly increased tGSK‐3β was also observed in the hippocampus of E4‐T2 mice (Figure 5e,h), which was further confirmed by immunohistochemistry (Figure 5c,d). These findings indicate that ApoE4 disrupts insulin signaling and enhances GSK‐3β activity in T2DM mice, which can contribute to the exacerbated AD‐related pathology in ApoE4‐T2DM populations.

### 3.5 Inhibiting Hippocampal GSK‐3β Alleviated the AD‐Like Pathologies and Cognitive Deficits in ApoE4‐T2DM Mice

**FIGURE 6:**
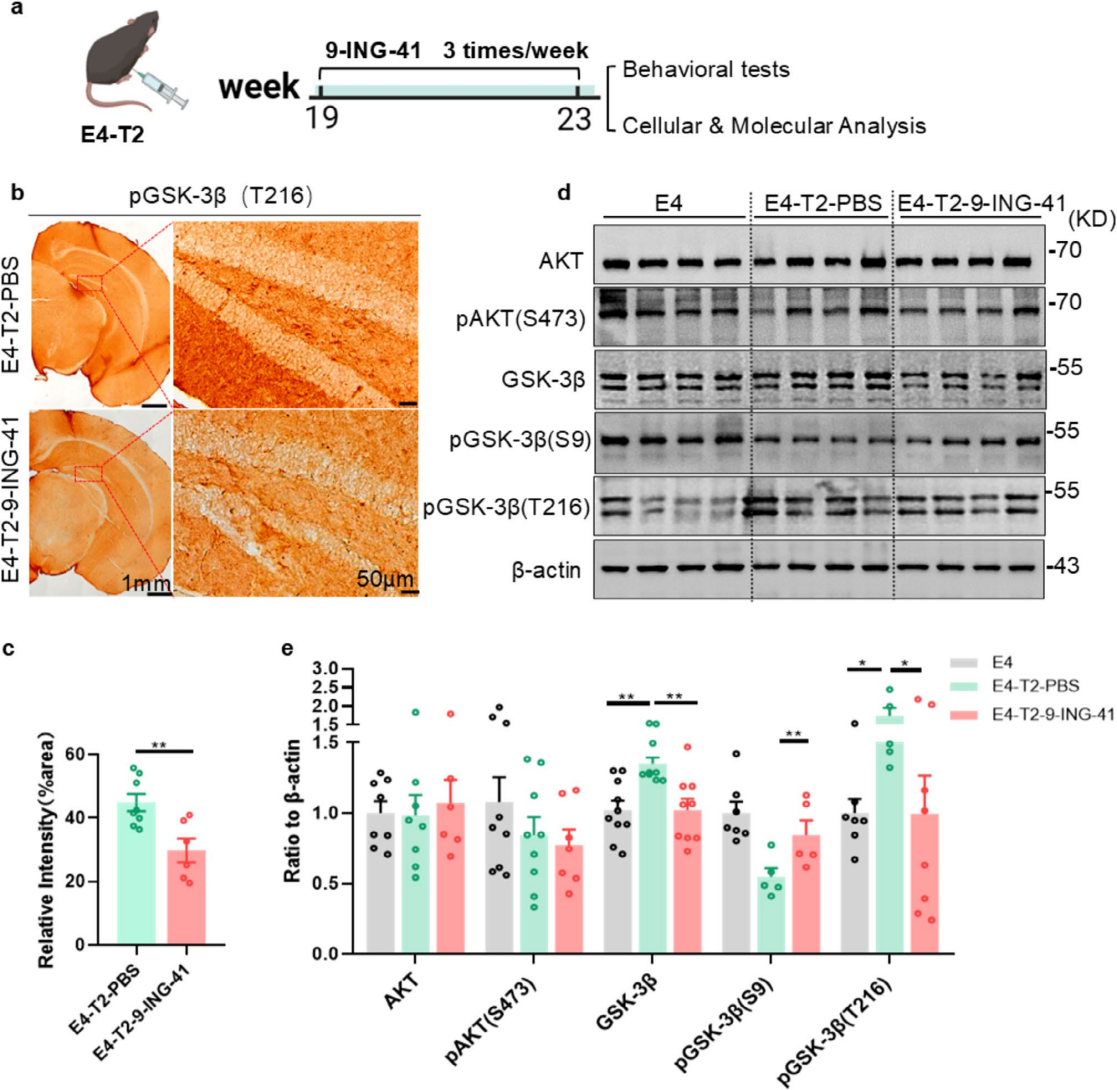
9‐ING‐41 reduced GSK‐3β levels in the hippocampus of ApoE4‐T2DM mice without affecting upstream insulin signaling. (a) Flowchart of the administration process for the GSK‐3β inhibitor (9‐ING‐41) administered intraperitoneally to ApoE4‐T2 mice (E4‐T2). (b) immunohistochemistry representative images of pGSK‐3β (Tyr‐216) in ApoE4‐T2 mice treated with PBS (E4‐T2‐PBS) and ApoE4‐T2 mice treated with 9‐ING‐41 (E4‐T2‐9‐ING‐41), along with (c) quantification of the results. Scale bar =50 μm, 6–7 mice per group, unpaired *t*‐test with Welch's correction. (d) Representative images of insulin signaling pathway protein expression in ApoE4 mice on a normal diet (E4), ApoE4‐T2 mice treated with PBS (E4‐T2‐PBS), and ApoE4‐T2 mice treated with 9‐ING‐41 (E4‐T2‐9‐ING‐41), along with (e) quantification of the western blotting results. Six to nine mice per group, one‐way ANOVA followed by Bonferroni's tests. Six‐month‐old mice, *p < 0.05, **p < 0.01, data presented as mean ± SEM.

To further evaluate the potential of GSK‐3β as a therapeutic target for AD‐like pathologies and cognitive impairments in ApoE4‐T2DM conditions, we administered intraperitoneally 9‐ING‐41, a specific small‐molecule inhibitor of GSK‐3β [45], into the ApoE4‐T2DM mice (Figure 6a). We observed that 9‐ING‐41 significantly inhibited GSK‐3β expression level with modulated phosphorylation status in the hippocampus of E4‐T2 mice (Figure 6d,e). Specifically, 9‐ING‐41 increased inhibitory phosphorylation of GSK‐3β at Ser9 and decreased the level of Tyr216 phosphorylation, the active form of GSK‐3β (Figure 6b–e). However, 9‐ING‐41 did not affect the expression or phosphorylation of AKT (Figure 6d,e), which serves as an upstream regulator in the insulin signaling pathway. Serum biochemical analysis indicated that the intraperitoneal injection of 9‐ING‐41 significantly reduced random blood glucose levels in E4‐T2 mice without causing changes in body weight (Figure S5a,b). Additionally, 9‐ING‐41 treatment induced a decreasing trend in TG and LDL in E4‐T2 mice (Figure S5g–j). Although 9‐ING‐41 did not affect liver index or liver transaminases (ALT and AST) in E4‐T2 mice (Figure S5d–f), the mouse livers treated with 9‐ING‐41 appeared redder, and the sensation of a surface oil film was notably reduced (Figure S5c). These data suggest that 9‐ING‐41 effectively downregulates GSK‐3β expression and activity in the hippocampus with a limited effect on glucose and lipid metabolism in the hippocampus of ApoE4‐T2DM mice.

**FIGURE 7:**
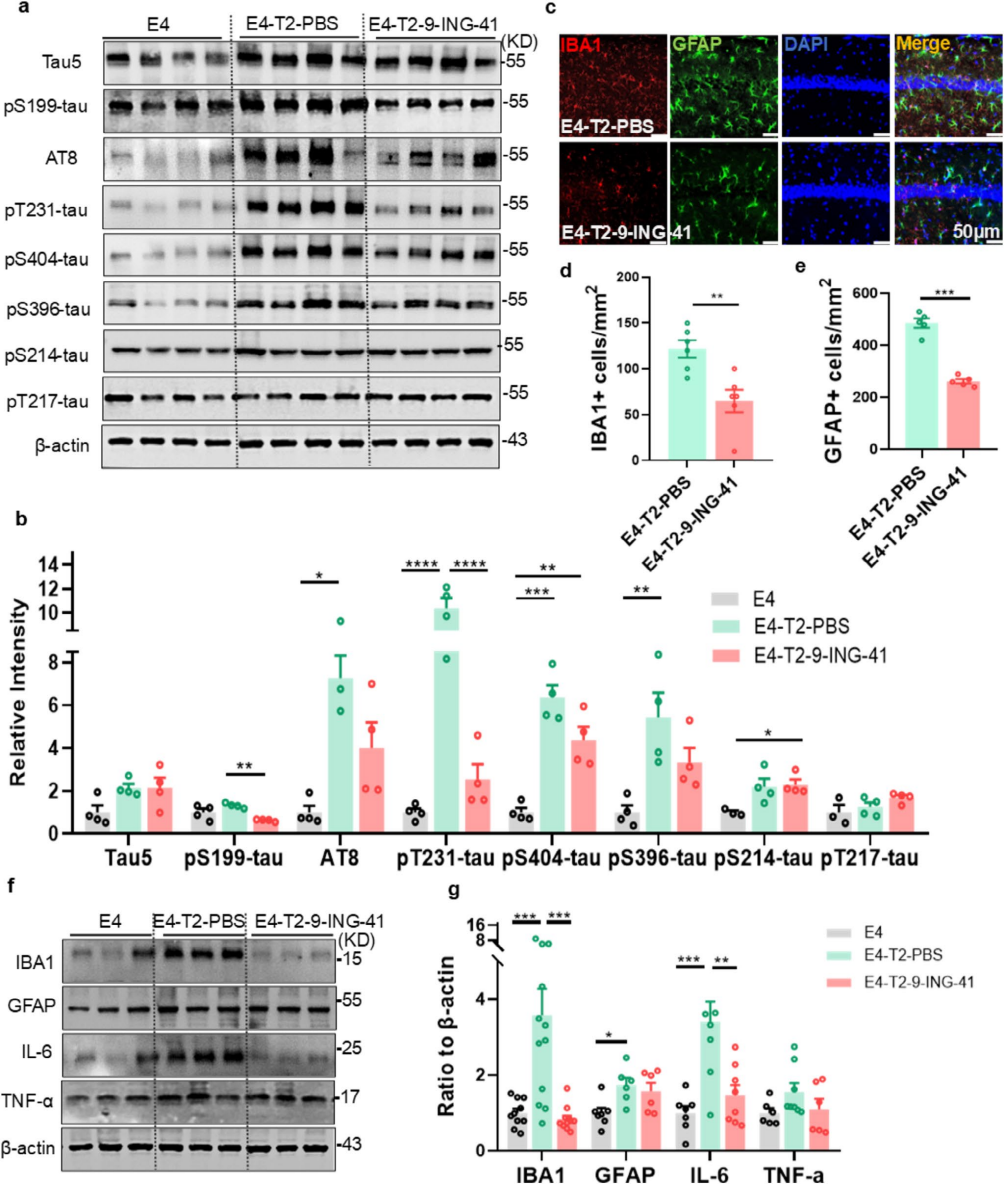
9‐ING‐41 reduced tau protein phosphorylation and ameliorated gliosis in the hippocampus of E4‐T2 mice. (a) Representative images of tau phosphorylation levels at multiple sites in the hippocampus of ApoE4 normal diet group (E4), ApoE4‐T2DM mice with intraperitoneal PBS injection group (E4‐T2‐PBS), and ApoE4‐T2DM mice with intraperitoneal 9‐ING‐41 injection group (E4‐T2‐9‐ING‐41). (b) Quantitative analysis of Western blotting results. Three to four mice per group, one‐way ANOVA followed by Bonferroni's tests. (c) Representative images of immunofluorescence staining for microglia and astrocytes in the CA1 of the hippocampus for each group of mice. (d) Microglial and (e) astrocyte counts in 1 mm^2^ area of the CA1 of the hippocampus. Scale bar = 50 μm, 5–6 mice per group, unpaired *t*‐test with Welch's correction. (f) Protein expression levels of glial cells and related inflammatory factors in hippocampus of mouse and (g) quantitative analysis. Six to ten mice per group, one‐way ANOVA followed by Bonferroni's tests. 6‐month‐old mice, *p < 0.05, **p < 0.01, ***p < 0.001, ****p < 0.0001, data expressed as mean ± SEM.

To determine whether AD‐like pathologies could be alleviated by intraperitoneal injection of 9‐ING‐41, we assessed its effects on tau phosphorylation, gliosis, and synaptic dysfunction in the hippocampus of T2DM mice. The phosphorylation levels of tau at Ser199, AT8 (Ser202/Thr205), Thr231, and Ser404 in the hippocampus of E4‐T2‐9‐ING‐41 mice were significantly reduced compared with those of PBS‐injected E4‐T2 (E4‐T2‐PBS) mice measured by Western blotting (Figure 7a,b). Additionally, 9‐ING‐41 mitigated the activation of microglia and astrocytes (Figure 7c–e), with significantly decreased levels of inflammatory mediators in the hippocampus measured by Western blotting and immunofluorescence (Figure 7a,b,f,g). These results suggest that GSK‐3β inhibitor 9‐ING‐41 alleviates AD‐like tau hyperphosphorylation and gliosis in ApoE4‐T2DM mice.

**FIGURE 8:**
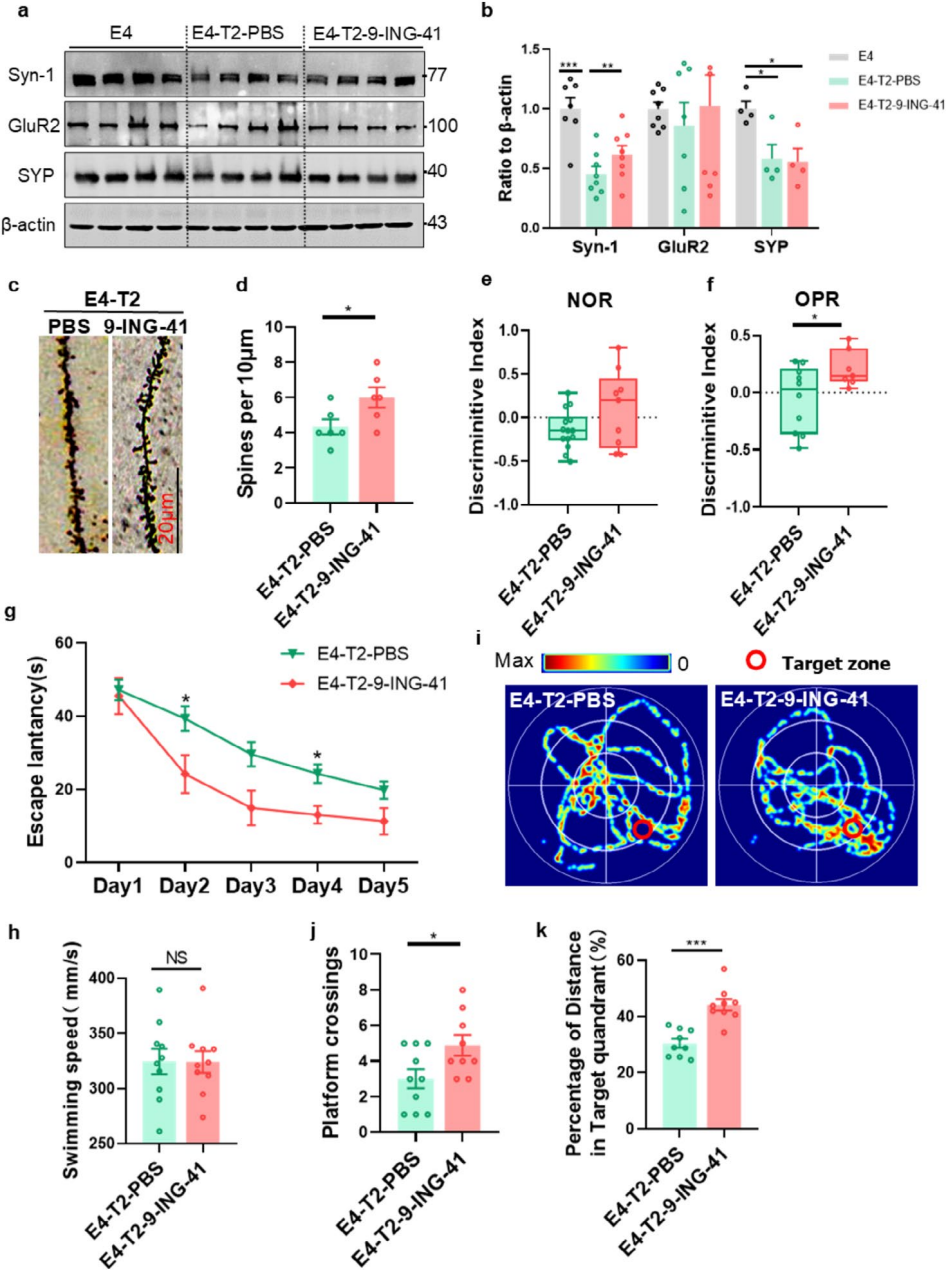
9‐ING‐41 rescued synaptic deficits and cognitive impairment in ApoE4‐T2DM mice. (a) Representative images of synaptic‐associated protein expression levels in the hippocampus of mice and (b) results of quantitative analysis. Four to seven mice per group, one‐way ANOVA followed by Bonferroni's tests. (c) Golgi staining results in the DG region of the mouse hippocampus and (d) comparison of dendritic spine counts of 10 μm length. Scale bar = 20 μm, six mice per group, unpaired *t*‐test with Welch's correction. (e, f) Discrimination index statistics from the NOR test and the OPR test. 9–12 mice per group, unpaired *t*‐test with Welch's correction. (g) Latency of mice during the learning phase of the MWM. (h) Average movement speed and (i) representative heat map of the movement trajectory in the MWM experiment. (j) Number of platform crossings and (k) percentage of distance traveled in the target quadrant for each group of mice in the MWM experiment. 9–10 mice per group, unpaired *t*‐test with Welch's correction. Six‐month‐old mice, *p < 0.05, **p < 0.01, ***p < 0.001, data expressed as mean ± SEM.

To evaluate the effects of intraperitoneal injection of 9‐ING‐41 on cognitive function and the molecular mechanisms in ApoE4‐T2DM mice, we observed that 9‐ING‐41 significantly increased Syn‐1 level without affecting GluR2 and synaptophysin (SYP) levels in the hippocampus of E4‐T2 mice (Figure 8a,b). Spine density was significantly increased in E4‐T2‐9‐ING‐41 mice compared with E4‐T2‐PBS mice (Figure 8c,d). Furthermore, E4‐T2 mice treated with 9‐ING‐41 showed an increased discrimination index compared with the PBS‐treated group in the NOR and OPR tests (Figure 8e,f). During the MWM training trials, E4‐T2‐9‐ING‐41 mice exhibited shorter latencies to find the platform on days 2 and 4 compared with the E4‐T2‐PBS group (Figure 8g). Consistently, 9‐ING‐41‐treated mice showed an increased number of platform crossings (Figure 8i,j) and a higher percentage of distance traveled in the target quadrant during the test phase (Figure 8k). Additionally, there were no significant differences in swimming speed (Figure 8h). Overall, these data suggest that inhibiting GSK‐3β by 9‐ING‐41 effectively mitigates synaptic deficits and cognitive impairment in ApoE4‐T2DM mice.

## 4 Discussion

T2DM and ApoE4 are recognized independent risk factors for neurodegenerative diseases, including AD, but the mechanisms by which they increase the risk of neurodegeneration remain unclear [46, 47]. In the present study, we revealed that ApoE4 intensified brain insulin signaling disruption with activated GSK‐3β, increased tau phosphorylation, increased Aβ42/Aβ40 ratio in cortex, enhanced gliosis and neuroinflammation, exacerbated synaptic damage and cognitive deficits in T2DM mice. Inhibiting GSK‐3β efficiently mitigated the AD‐like pathologies and cognitive impairments in the ApoE4‐T2DM mice.

Diabetes mellitus (DM) is a prevalent metabolic disorder that poses a significant public health challenge. Epidemiological studies predict that approximately 643 million individuals worldwide will be affected by DM by 2030 [48]. T2DM has been identified as an independent risk factor for AD [47]. ApoE4 is the most common genetic risk factor for AD, playing a critical role in the regulation of Aβ transport and clearance, thus making it a vital therapeutic target for AD [49]. This study investigated how the ApoE4 isoform exacerbated AD‐related cognitive impairment in the context of T2DM. Given that rodents have only one ApoE genotype, which contains the equivalents of Arg‐112 and Glu‐255, similar to human ApoE4 [31, 32], we employed humanized ApoEε3/ε3 and ApoEε4/ε4 knock‐in mice. In these models, mouse ApoE ε2, 3, and a majority of ε4 were replaced with human ApoE gene sequences, including portions of the 3′ untranslated region [29]. These mice were subjected to a high‐fat diet (HFD) in conjunction with low‐dose multiple intraperitoneal injections of streptozotocin (STZ) to model T2DM [30]. HFD feeding induced obesity and mild hyperglycemia in mice, mimicking the insulin resistance state observed in pre‐diabetic patients. Low‐dose STZ injections resulted in β‐cell ablation in the pancreas, leading to dysfunction in compensatory insulin secretion and resultant hyperglycemia in the animals [50]. This chemical induction method for T2DM modeling is cost‐effective, easy to implement, and more accurately reflects the disease processes and characteristics of human T2DM [34]. Our study provided fundamental reference information for constructing and validating animal models of SAD. We found that both T2DM and ApoE4 promote AD‐like pathologies, including tau pathology, gliosis, and synaptic damage. Furthermore, the combined effects of ApoE4 and T2DM were greater than those of each factor alone, suggesting that early prevention and intervention for AD should prioritize high‐risk populations, particularly T2DM patients carrying the ApoE4 genotype.

GSK‐3, consisting of GSK‐3α and GSK‐3β isoforms, is a serine/threonine kinase that plays a pivotal role in glucose metabolism [51]. GSK‐3β, predominantly expressed in the liver, muscle, adipose tissue, and brain, is critically involved in the pathogenesis of diabetes, neurodegenerative diseases, and chronic inflammatory conditions [52]. Elevated GSK‐3β activity in the brains of AD patients leads to tau hyperphosphorylation and abnormal aggregation of Aβ, thereby accelerating the progression of AD [53]. GSK‐3β is an essential component of the insulin signaling pathway and is integral to glycogen metabolism [54]. ApoE4 has been shown to promote peripheral metabolic disturbances, including dyslipidemia and systemic insulin resistance. For example, ApoE4 carriers often exhibit elevated plasma levels of low‐density lipoprotein (LDL) and triglycerides (TG), which are associated with increased inflammation and endothelial dysfunction [55, 56]. These metabolic alterations may compromise blood–brain barrier integrity and contribute to neuroinflammation, thereby facilitating the development of CNS pathology. Moreover, peripheral insulin resistance can lead to impaired nutrient sensing and systemic inflammation, which have been implicated in the propagation of central insulin resistance, which is a key feature in AD [57, 58]. In T2DM, impaired insulin signaling hinders upstream AKT from phosphorylating the Ser9 of GSK‐3β, resulting in increased GSK‐3β activity that promotes AD‐like pathologies [21, 22, 23]. Research by Bu's team demonstrated that ApoE4 impaired cerebral insulin signaling in an age‐dependent manner in vivo [44]. Our study corroborated that ApoE4 exacerbated insulin signaling deficits in T2DM mice, specifically by inhibiting AKT Ser473 phosphorylation and reducing GSK‐3β Ser9 phosphorylation. Furthermore, ApoE4 upregulated GSK‐3β and enhanced its phosphorylation at Thr216, a key activation site. The activation or increased expression of GSK‐3β induced by ApoE4 further exacerbates tau hyperphosphorylation, neuroinflammation, and synaptic damage in T2DM mice, ultimately leading to significant impairments in learning and memory.

Although the ApoE4‐T2DM (E4‐T2) mice exhibit the most pronounced AD‐related pathological alterations, we did not observe significant loss of mature neurons in this cohort. We speculate that the absence of substantial neuronal loss reflects an early stage of AD pathology, during which endogenous neuroprotective and compensatory mechanisms such as synaptic plasticity, repair processes, and regenerative responses are still active [59, 60]. Furthermore, tau hyperphosphorylation may serve a protective role by stabilizing β‐catenin and delaying apoptosis, thereby preserving neuronal integrity at this stage [61, 62]. We observed an increased Aβ42/Aβ40 ratio in the cortex of ApoE4‐T2DM mice through ELISA, even though Thioflavin S staining did not reveal visible amyloid plaques. This “pre‐plaque” phase is increasingly recognized as a critical window for pathogenic activity, as soluble Aβ oligomers are considered more neurotoxic than fibrillar deposits and are capable of inducing synaptic dysfunction, network instability, and cognitive deficits prior to plaque formation [63, 64]. Therefore, our findings highlight the importance of monitoring early amyloid dynamics and suggest that therapeutic interventions targeting oligomer formation or clearance may be particularly effective at this stage. The direct regulatory relationship between ApoE4 and GSK‐3β remains an active area of investigation. Notably, studies have indicated that ApoE4 impairs insulin signaling upstream of GSK‐3β, potentially through dysregulation of PI3K‐dependent Akt activation, which in turn leads to reduced inhibitory phosphorylation of GSK‐3β at Ser9 [44]. In parallel, the Erk (extracellular signal‐regulated kinase) pathway has also been shown to influence GSK‐3β activity, suggesting that ApoE4 may engage multiple signaling axes to modulate its downstream effects [65]. Specifically, ApoE4 may interfere with PI3K‐Akt signaling by altering receptor interactions, such as with low‐density lipoprotein receptor‐related protein 1 (LRP1) or the low‐density lipoprotein receptor (LDLR), leading to impaired Akt Ser473 phosphorylation and subsequent loss of GSK‐3β inhibition [44, 66]. Additionally, ERK‐mediated phosphorylation of GSK‐3β at Thr43 may further regulate its substrate specificity and enzymatic activity [65, 67], although the exact interplay between these pathways in the context of ApoE4 remains unclear. Understanding how ApoE4 engages these signaling cascades will provide critical insights into its role in AD pathogenesis. Our data support these findings by showing significant changes in GSK‐3β phosphorylation levels in ApoE4‐T2DM mice, highlighting the critical role of ApoE4 in mediating AD‐related pathological changes and cognitive impairments via GSK‐3β. However, the exact molecular mechanisms underlying this regulation are not fully understood. Future studies should aim to identify specific binding sites or post‐translational modifications that mediate the interaction between ApoE4 and GSK‐3β and could also include in vitro assays using purified proteins to further explore the direct effects of ApoE4 on GSK‐3β activity. Additionally, while our findings emphasize the role of insulin signaling, investigating contributions from alternative pathways, like Wnt/β‐catenin, will be necessary to comprehensively understand how ApoE4 modulates GSK‐3β activity.

A variety of GSK‐3 inhibitors such as lithium and synthetic ATP‐competitive small molecules, including SB‐216763, have been extensively researched for the treatment of neurodegenerative conditions and psychiatric disorders [68, 69, 70]. The novel compound CHIR98023 has been demonstrated to improve insulin resistance in diabetic mouse models and presents promise as a therapeutic intervention for T2DM [71]. In selecting 9‐ING‐41 (Elraglusib) for our study, we considered its unique profile as a reversible ATP‐competitive GSK‐3 inhibitor that has shown significant inhibition of GSK‐3β activity and preclinical antitumor activity in several cancers, including pancreatic and bladder cancers [45, 72, 73]. Notably, 9‐ING‐41 was recently granted fast‐track designation by the U.S. Food and Drug Administration (FDA) for the treatment of patients with pancreatic cancer [74], highlighting its translational potential. It has also advanced into Phase I clinical trials in oncology, demonstrating acceptable safety and pharmacokinetic profiles in human subjects [72]. 9‐ING‐41was identified from a library of GSK‐3 inhibitors specifically optimized for high blood–brain barrier penetration, suggesting its ability to exert central nervous system effects. Compared with other GSK‐3 inhibitors, 9‐ING‐41 offers several advantages. Unlike lithium, which requires careful monitoring due to its narrow therapeutic window and potential side effects, 9‐ING‐41 demonstrates higher specificity and lower toxicity [68]. Additionally, while CHIR98023 primarily targets insulin resistance [71], the broader mechanism of action of 9‐ING‐41 may confer more comprehensive benefits in mitigating AD‐related cognitive impairment in T2DM. However, its potential to mitigate AD‐related cognitive impairment in T2DM has not yet been explored. The dosage of 9‐ING‐41 was chosen as 20 mg/kg based on literature reports indicating its efficacy and safety profile [45, 73]. Our data further confirmed that intraperitoneal administration of 9‐ING‐41 at this dosage is effective to alleviate AD‐like pathologies and enhance learning and memory functions. Moreover, 9‐ING‐41 mitigated the adverse effects of ApoE4 on the progression from T2DM to AD through multiple mechanisms and underscores the pivotal role of GSK‐3β in this process. Our current findings do not exclude the potential contribution of peripheral metabolic improvements to cognitive enhancement. Notably, 9‐ING‐41 treatment induced a decreasing trend in TG and LDL levels in ApoE4‐T2DM mice, suggesting its influence on peripheral metabolism. While our results demonstrate that 9‐ING‐41 can directly inhibit GSK‐3β activity in the hippocampus, it remains unclear whether and how peripheral metabolic changes might affect central nervous system function. Clarifying these interactions will be essential for understanding the full therapeutic potential of 9‐ING‐41. Consequently, 9‐ING‐41 showed promise as an early preventive and therapeutic strategy for individuals at risk of developing AD. However, these findings need to be further validated through rigorous preclinical studies assessing blood–brain barrier penetration, target engagement in the brain, and safety before progressing to clinical trials to determine its efficacy and tolerability in human patients.

Together, we demonstrated that ApoE4 exacerbated cerebral AD‐like pathologies and cognitive impairments in T2DM mice by modulating insulin signaling pathways. The GSK‐3β inhibitor 9‐ING‐41 showed promise as a potential therapeutic for T2DM‐related cognitive decline. Future studies should explore the mechanisms by which the interaction between ApoE4 and T2DM contributed to AD pathology and evaluate the impact of GSK‐3β inhibitors on cognitive impairments in T2DM populations. Additionally, understanding the bidirectional interactions between the peripheral and central nervous systems regarding cognitive impairments in diabetes is a critical area for future research.

## Author Contributions

J.Z.W., Y.Z., and Y.G. designed the research; Y.Y.W., Y.G., and R.Y.W. performed the experiments. Y.Y.W., Q.F.Z., F.S., X.W., and J.X. performed the statistical analysis and data interpretation. Z.X., J.Z.Z., and H.X. provided valuable advice on this research. Y.Y.W., Y.G., and J.Z.W. wrote the manuscript. All authors have read and approved the final manuscript. J.Z.W. is the supervisor of this work, with full access to all the data in the study, and takes responsibility for the data integrity and accuracy.

## Ethics Statement

Protocols involving transgenic mice were approved by the Institutional Animal Care and Use Committee of Tongji Medical College, Huazhong University of Science and Technology.

## Conflicts of Interest

The authors declare no conflicts of interest.

## Supporting information


**Figure S1:** Type 2 diabetic mice show a consistently elevated blood glucose level with impaired lipid metabolism in both ApoE3 and ApoE4 genotypes.
**Figure S2:** ApoE4 showed limited effect on Aβ pathology in T2DM mice.
**Figure S3:** Neither T2DM nor ApoE4 caused neuronal loss.
**Figure S4:** Correlation between hippocampal tau phosphorylation and spatial memory performance.
**Figure S5:** Inhibiting GSK‐3β by intraperitoneal injecting 9‐ING‐41 ameliorated dysregulation of glucose in ApoE4‐T2DM mice.
**Table S1:** Antibodies and reagents used in this study.

**Data S1:** cns70575‐sup‐0002‐DataS1.docx.

## Data Availability

Data available on request from the authors. The data that support the findings of this study are available from the corresponding author upon reasonable request.
