# Supplementary figures and images for: ApoE4 Upregulates GSK‐3β to Aggravate Alzheimer‐Like Pathologies and Cognitive Impairment in Type 2 Diabetic Mice
Source: CNS Neurosci Ther. 2025 Sep 4;31(9):e70575. doi: 10.1111/cns.70575 (PMC12409304; doi:10.1111/cns.70575)

Figure 2

c

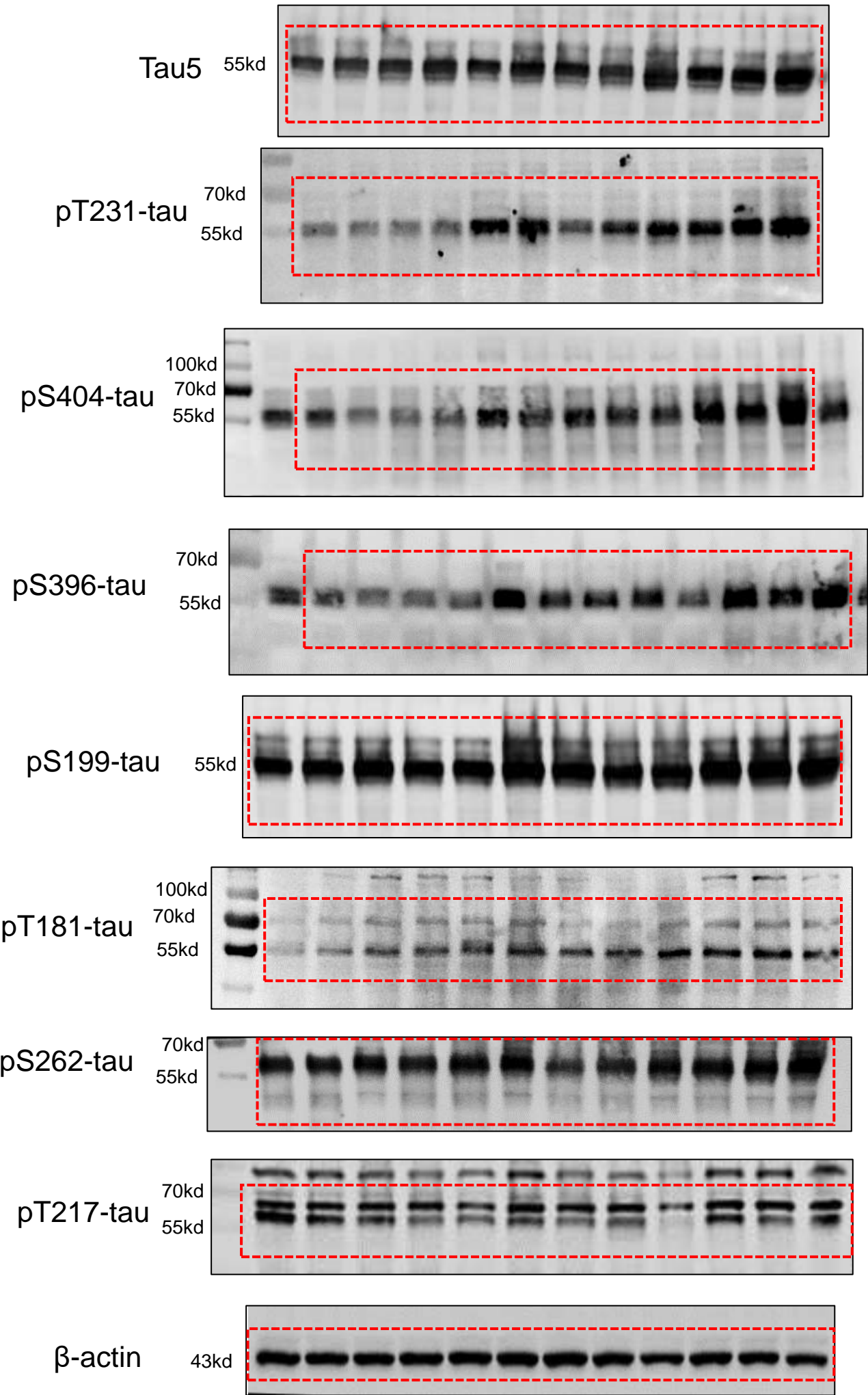

### Figure 3

**d**

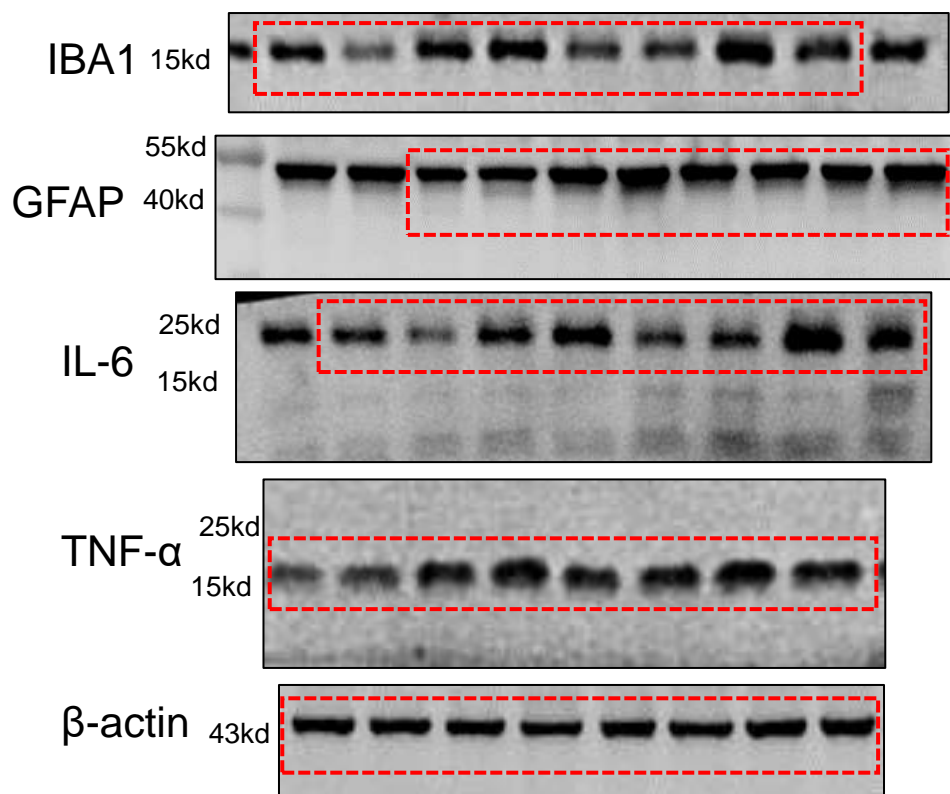**f**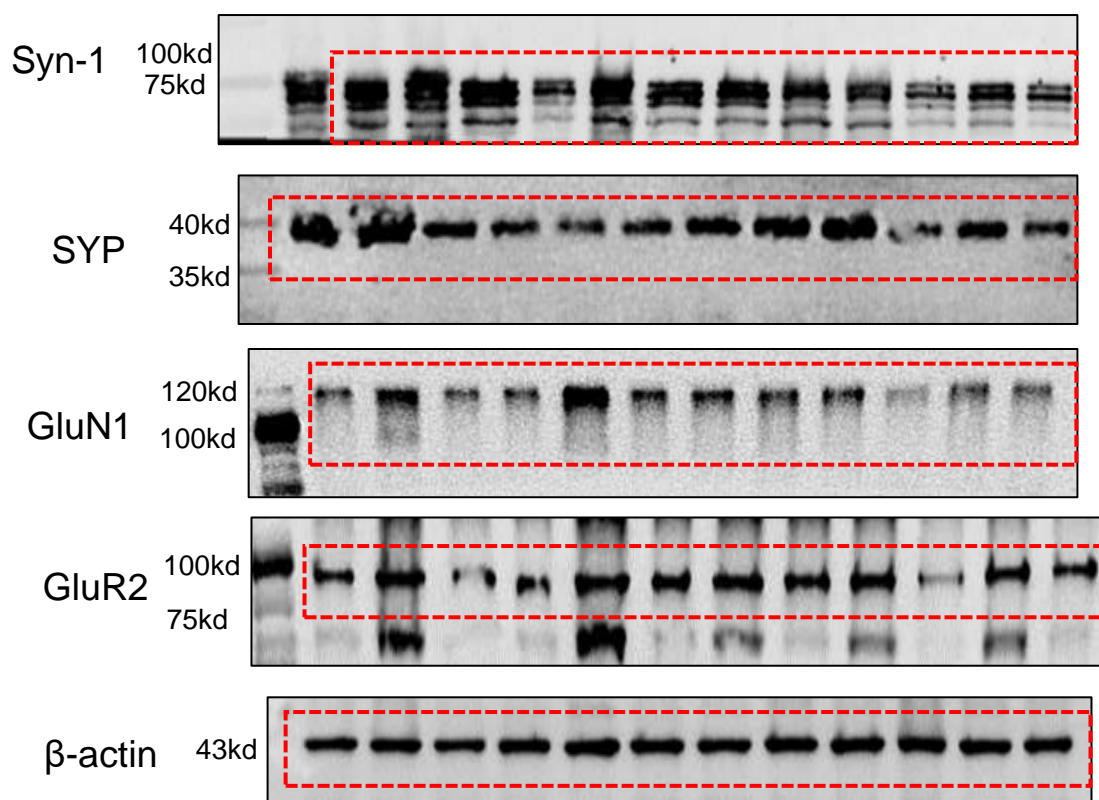

Figure 5

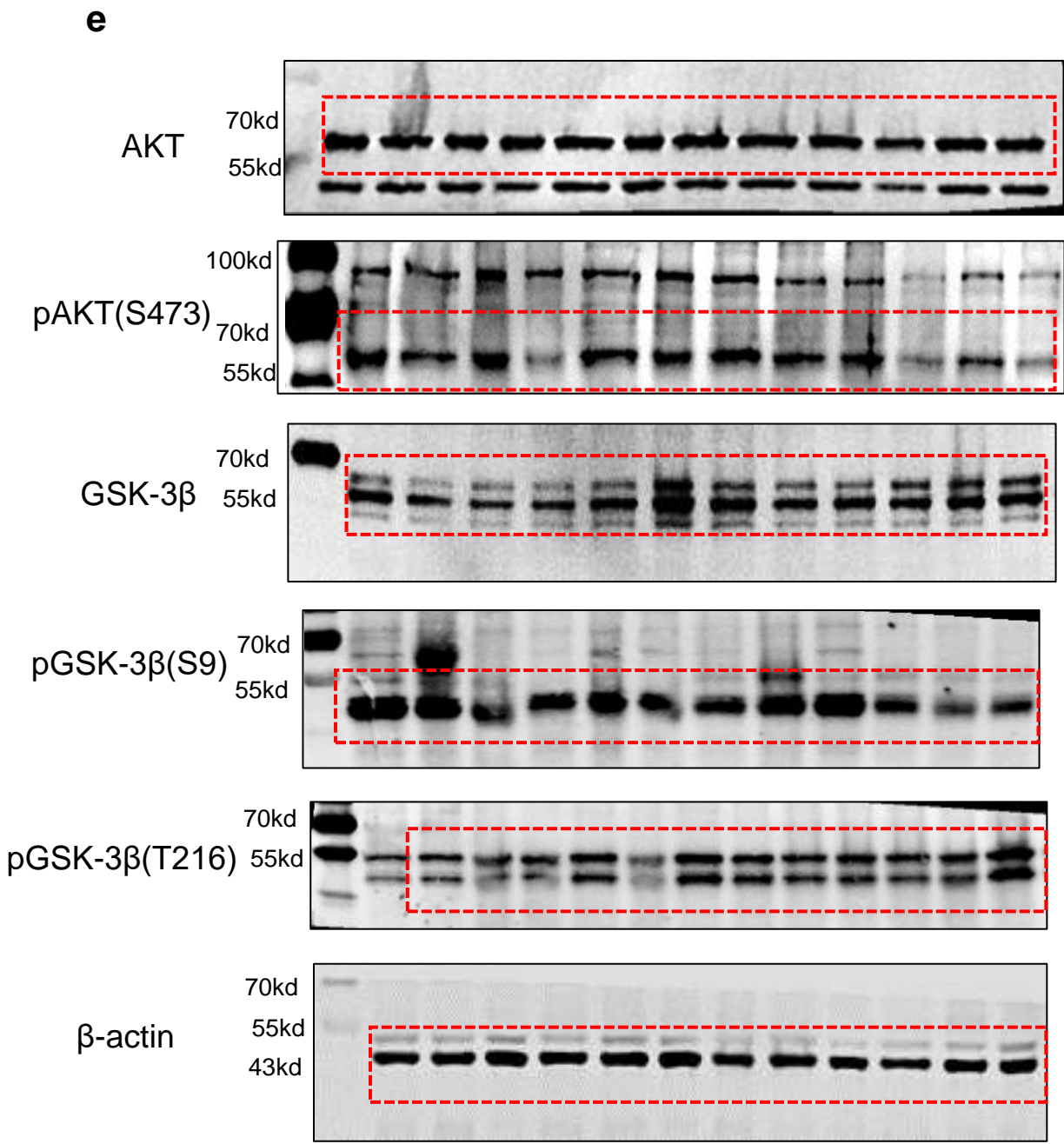

Figure 6

d

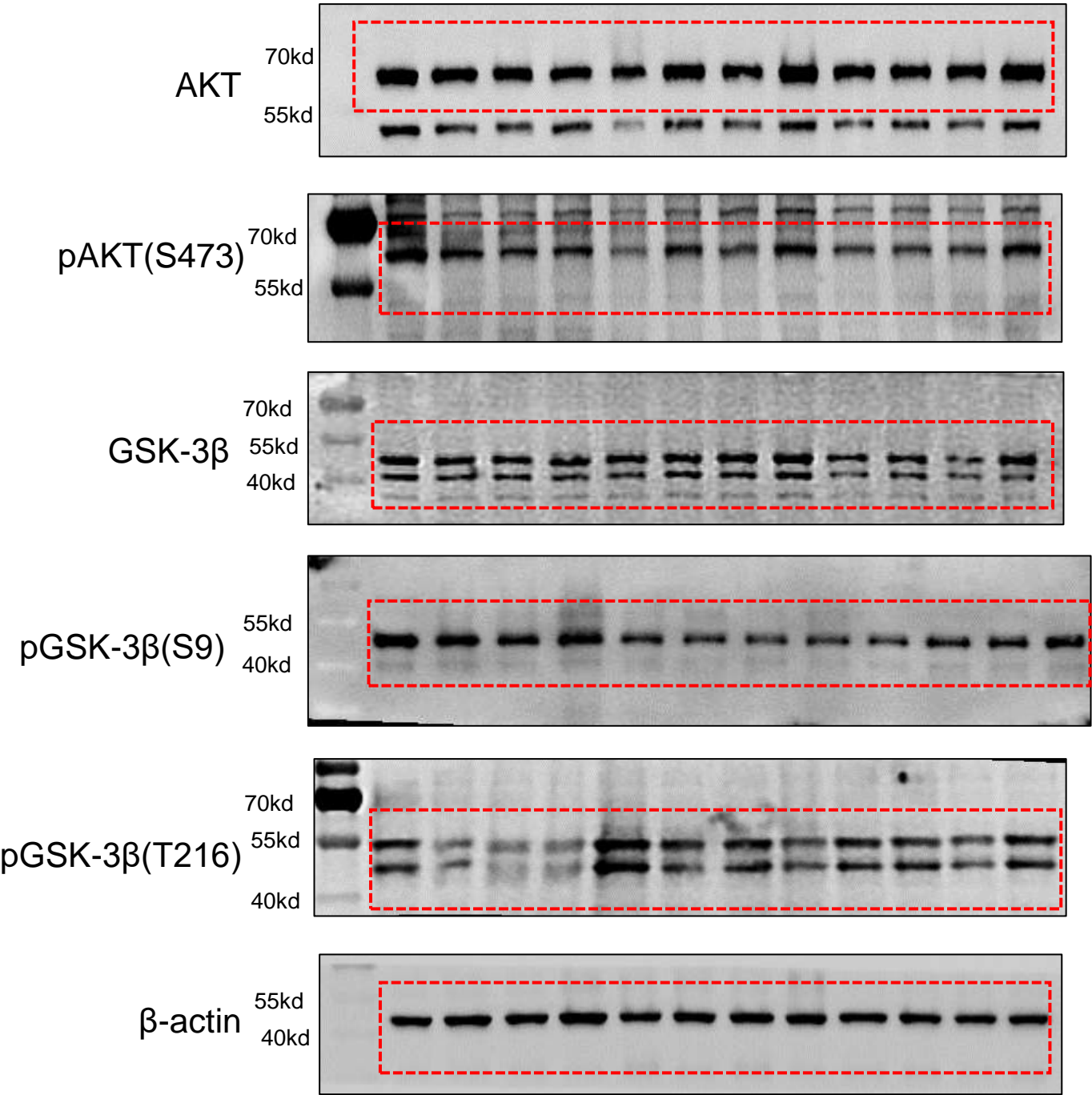

a

Figure 7

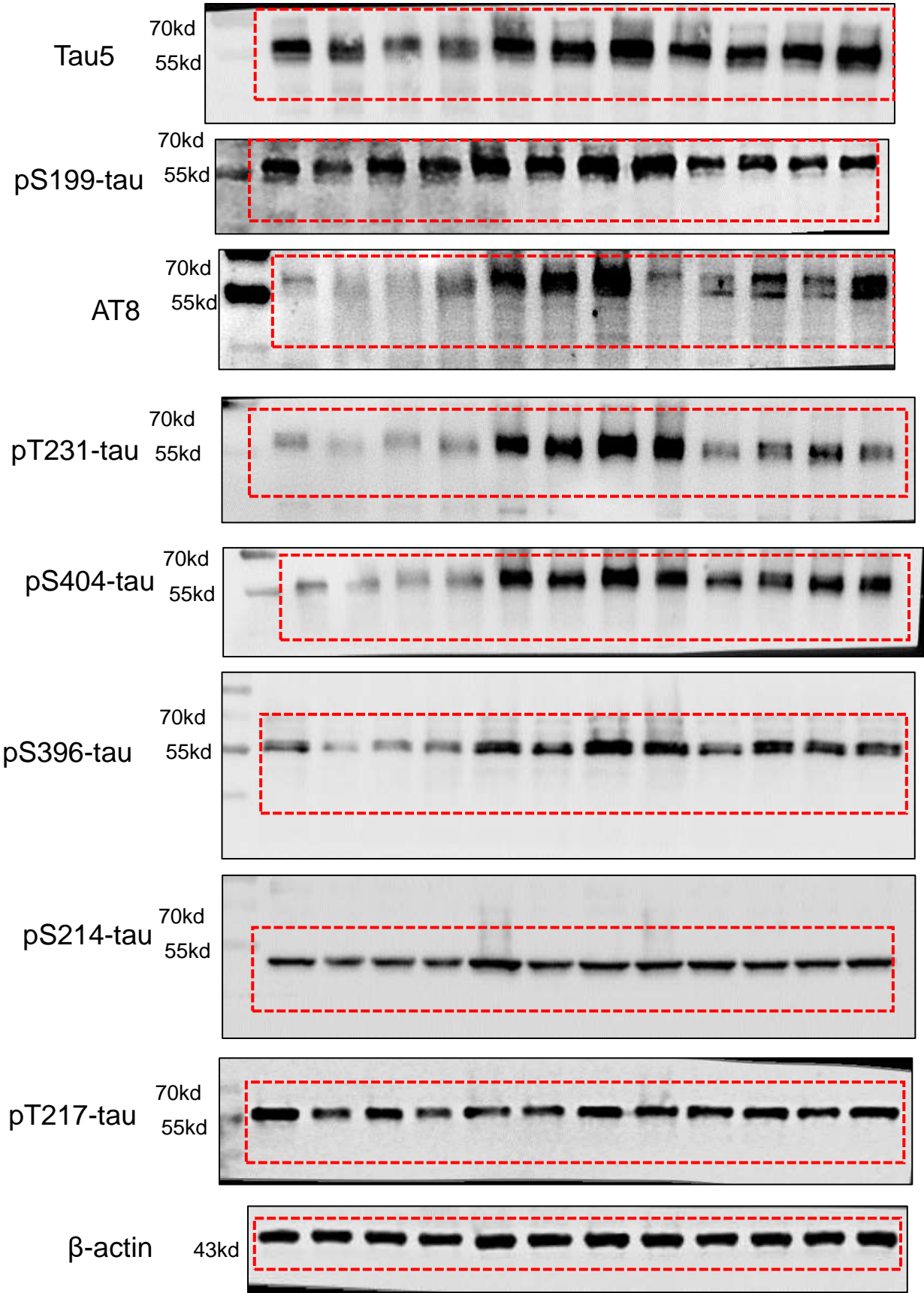

Figure 7

f

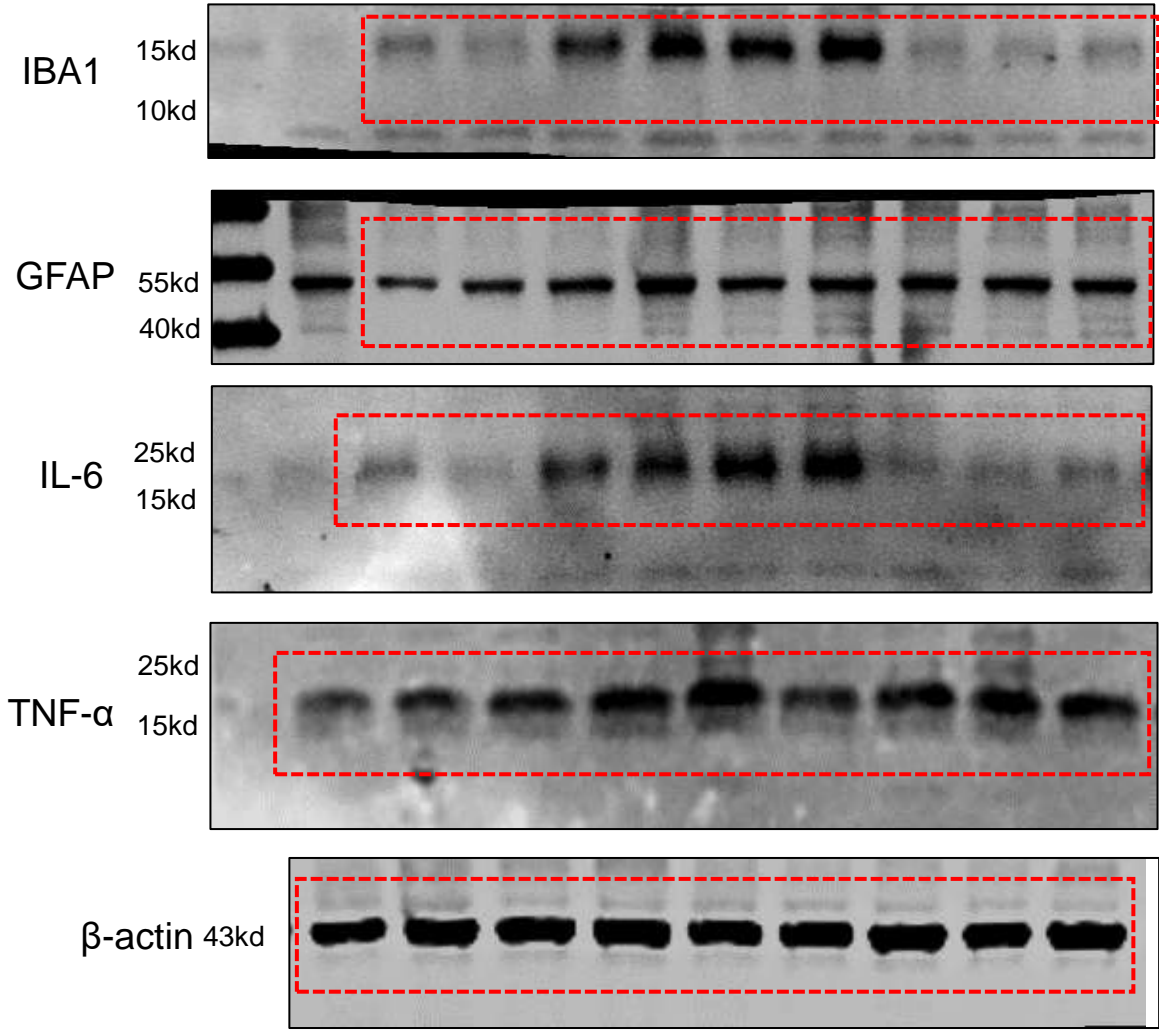

Figure 8

a

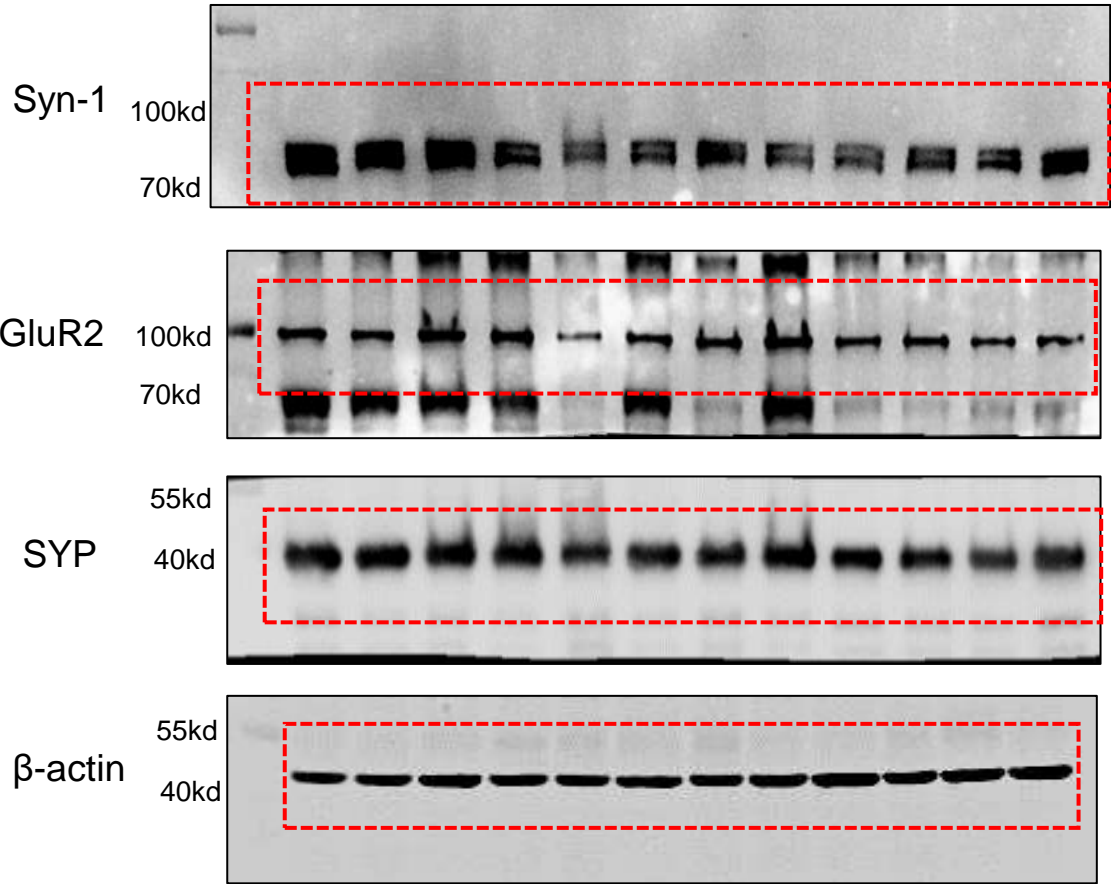

Supplement: Supplementary file 1 — Figure S1: Type 2 diabetic mice show a consistently elevated blood glucose level with impaired lipid metabolism in both ApoE3 and ApoE4 genotypes. Figure S2: ApoE4 showed limited effect on Aβ pathology in T2DM mice. Figure S3: Neither T2DM nor ApoE4 caused neuronal loss. Figure S4: Correlation between hippocampal tau phosphorylation and spatial memory performance. Figure S5: Inhibiting GSK‐3β by intraperitoneal injecting 9‐ING‐41 ameliorated dysregulation of glucose in ApoE4‐T2DM mice. Table S1: Antibodies and reagents used in this study. [file CNS-31-e70575-s001.pdf]
