# Supplementary material for: ApoE4 Upregulates GSK‐3β to Aggravate Alzheimer‐Like Pathologies and Cognitive Impairment in Type 2 Diabetic Mice
Source: CNS Neurosci Ther. 2025 Sep 4;31(9):e70575. doi: 10.1111/cns.70575 (PMC12409304; doi:10.1111/cns.70575)
Supplement: Supplementary file 2 — Data S1: cns70575‐sup‐0002‐DataS1.docx. [file CNS-31-e70575-s002.docx]

**Supplementary figures and tables**


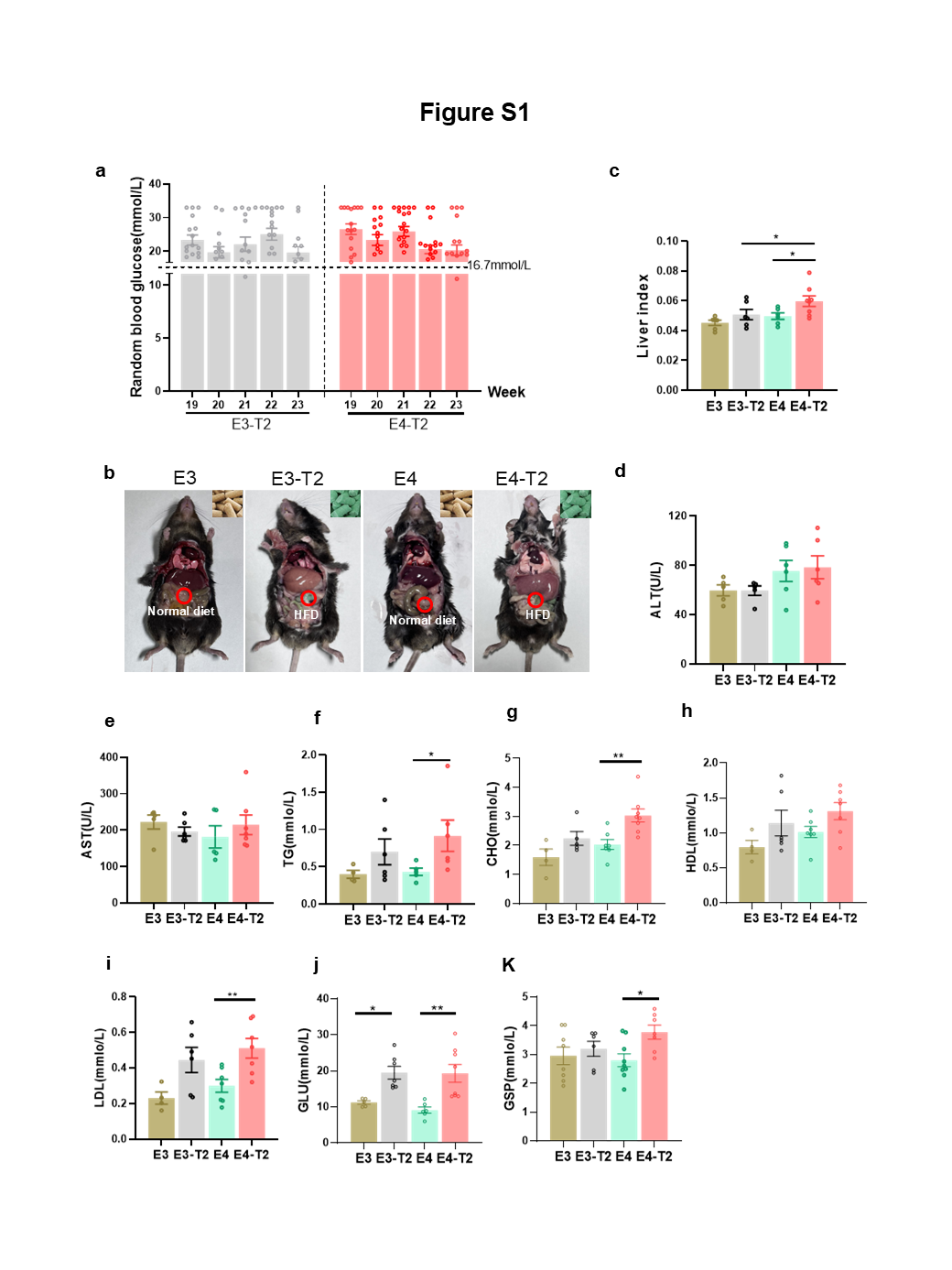


**Fig. S1 Type 2 diabetic mice show a consistently elevated blood glucose level with impaired lipid metabolism in both ApoE3 and ApoE4 genotypes.**

**(a)** The random blood glucose during the modeling period. E3-T2, n=16. E4-T2, n=18. **(b)** Representative macroscopic appearance of the liver and **(c)** Liver index (liver weight/body weight) statistical analysis. 6 mice per group, two-way ANOVA followed by Bonferroni's tests. **(d)** Serum alanine aminotransferase (ALT) comparison and **(e)** serum aspartate aminotransferase (AST) comparison in mice. 4-6 mice per group, two-way ANOVA followed by Bonferroni's tests. **(f-i)** The triglycerides (TG), cholesterol (CHO), high-density lipoprotein (HDL), and low-density lipoprotein (LDL) statistical analysis. **(j)** Serum glucose (GLU) comparison and **(k)** glycated serum protein (GSP) comparison in mice. 4-7 mice per group, two-way ANOVA followed by Bonferroni's tests. 6-month-old mice, ** p < 0.05, ** p < 0.01,* data presented as Mean ± SEM.

**
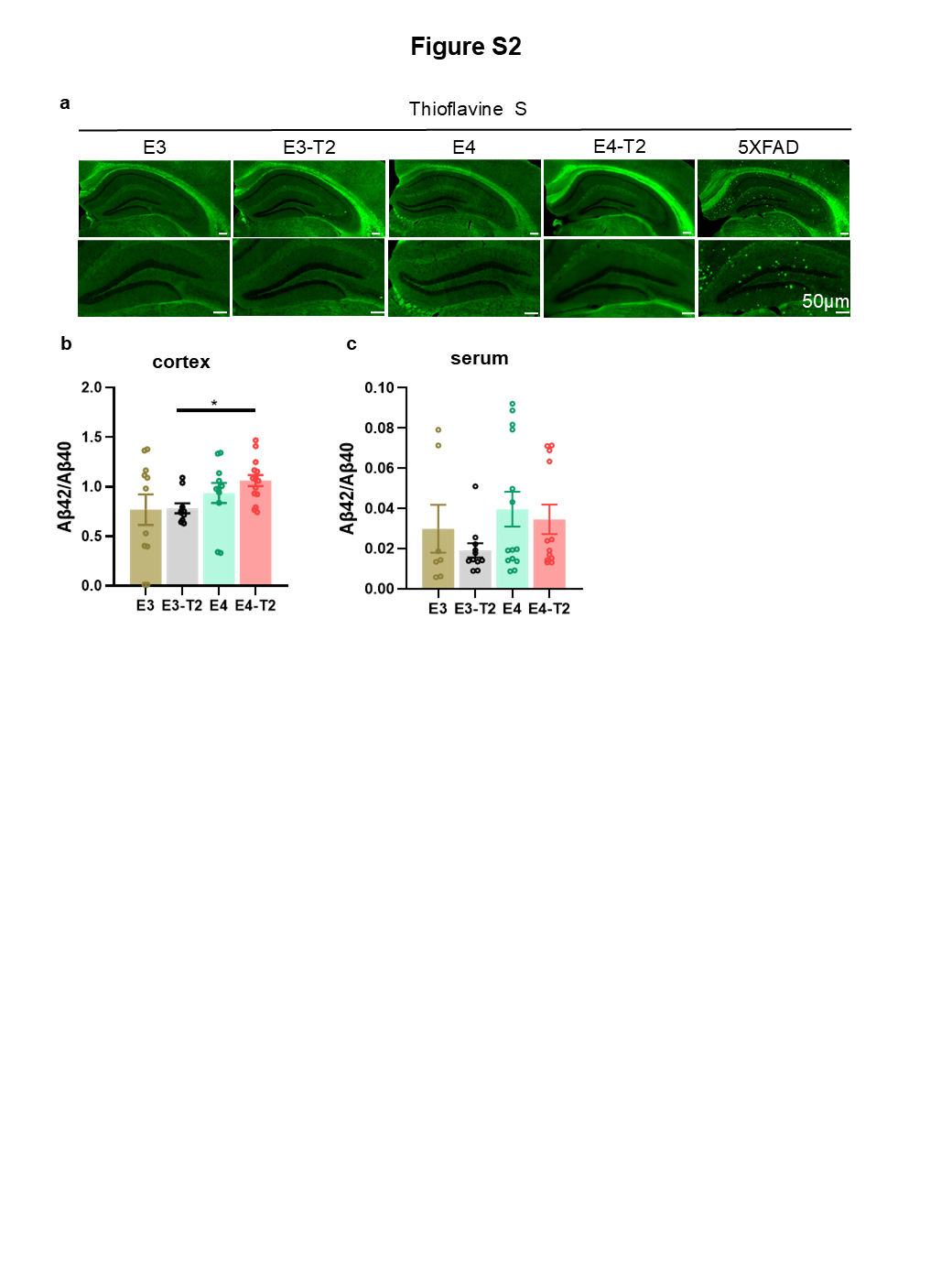
**

**Fig. S2 ApoE4 showed limited effect on Aβ pathology in T2DM mice.**

**(a)** Representative images of Thioflavin S staining in the hippocampus of each group of mice. Scale bar=50μm. **(b-c)** Enzyme-linked immunosorbent assay (ELISA) results and statistical analysis of and Aβ42/Aβ40 ratio in the cortex and serum of mice. 10-15 mice per group, two-way ANOVA followed by Bonferroni's tests. 6-month-old mice, ** p < 0.05,* data presented as Mean ± SEM.


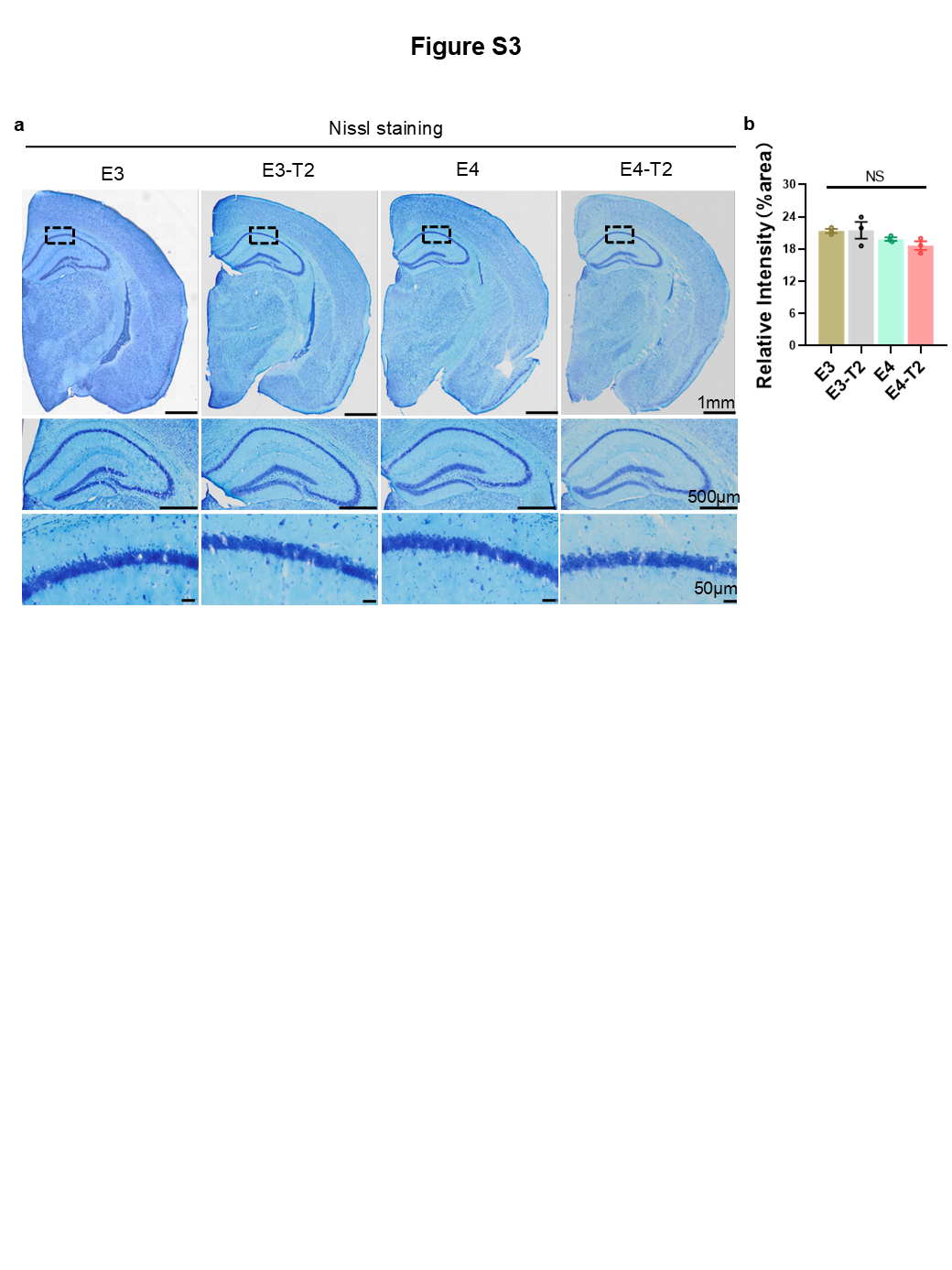


**Fig. S3 Neither T2DM nor ApoE4 caused neuronal loss.**

**(a)** Representative images of Nissl staining in various groups of mice and **(b)** the percentage of Nissl-positive stained area in the CA1 region. 3 mice per group, two-way ANOVA followed by Bonferroni's tests. 6-month-old mice, data presented as Mean ± SEM.


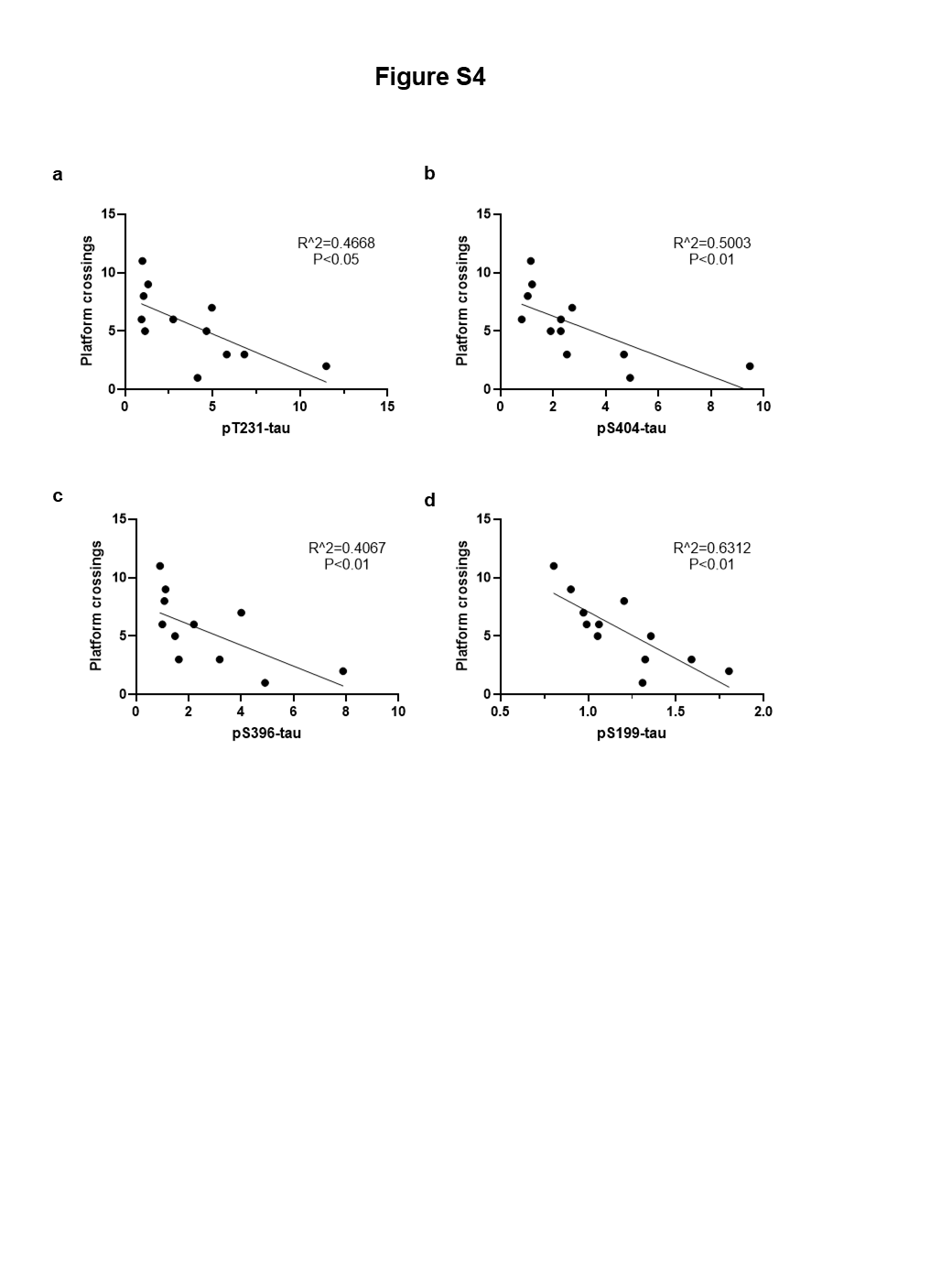


**Fig. S4 Correlation between Hippocampal Tau Phosphorylation and Spatial Memory Performance**

**(a)** Correlation between pT231-tau phosphorylation levels and platform crossings in the Morris water maze probe trial. **(b)** Correlation between pS404-tau phosphorylation levels and platform crossings in the Morris water maze probe trial. **(c)** Correlation between pS396-tau phosphorylation levels and platform crossings in the Morris water maze probe trial. **(d)** Correlation between pS199-tau phosphorylation levels and platform crossings in the Morris water maze probe trial. n=12 (3 mice per group, four groups: E3, E3-T2, E4, E4-T2), 6-month-old mice, data presented as Mean ± SEM.

**
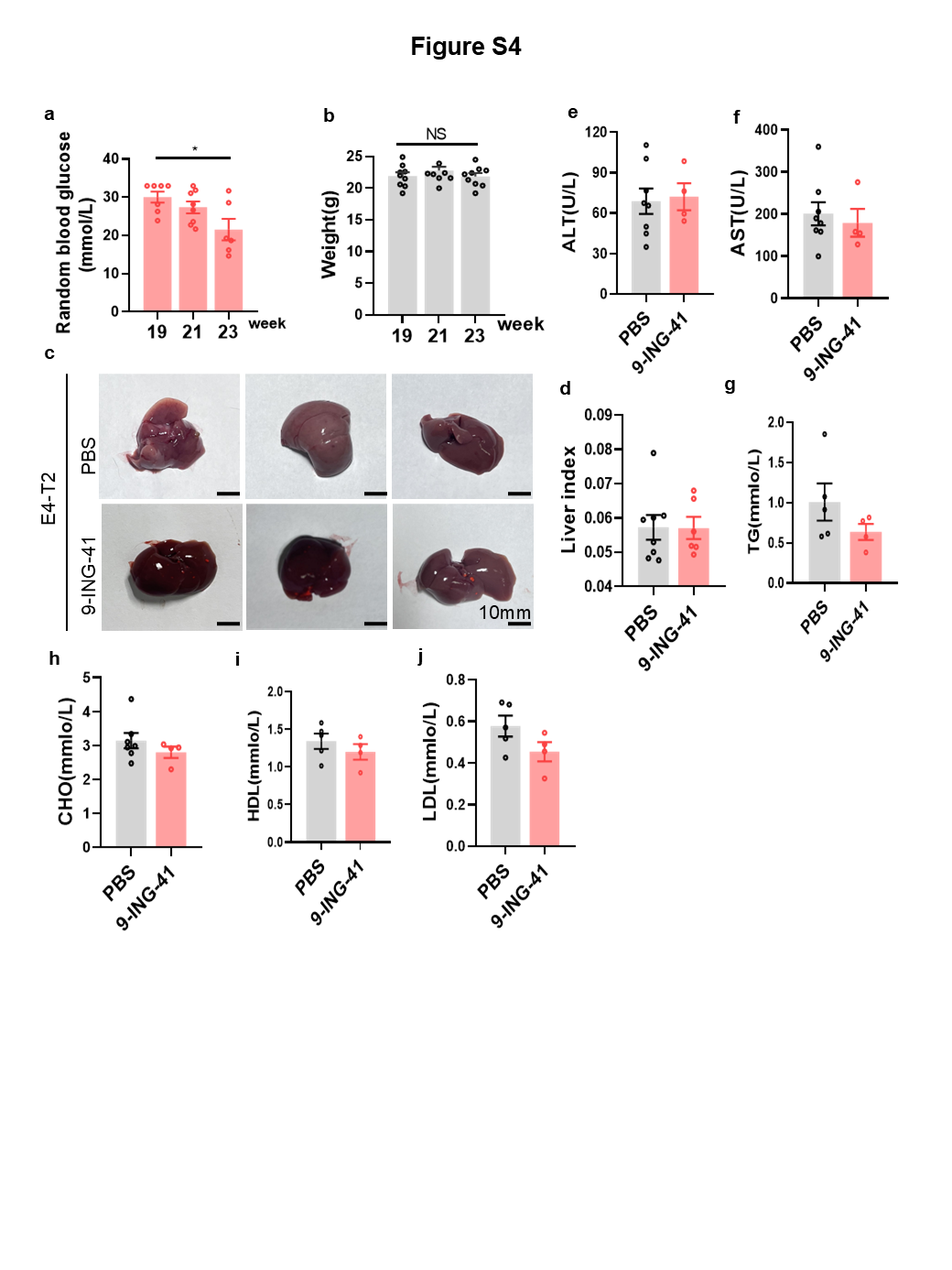
**

**Fig. S5 Inhibiting GSK-3β by intraperitoneal injecting 9-ING-41 ameliorated dysregulation of glucose in ApoE4-T2DM mice.**

**(a)** Random blood glucose variations in mice during intraperitoneal injection of 9-ING-41 and **(b)** body weight changes. 7-9 mice per group, unpaired t test with Welch’s correction. **(c)** Representative gross comparison of livers from PBS-injected mice (E4-T2-PBS) and 9-ING-41-injected mice (E4-T2-9-ING-41). **(d)** Comparison of liver index (liver weight/body weight) in mice. Scale bar =10mm, 6-8 mice per group, unpaired t test with Welch’s correction. **(e)** Statistical comparison of serum ALT and **(f)** AST levels in mice. **(g-j)** Statistical comparisons of TG, CHO, HDL, and LDL concentrations in mice. 4-8 mice per group, unpaired t test with Welch’s correction. 6-month-old mice, ** p < 0.05*, data presented as Mean ± SEM.

**Table S1. Antibodies and reagents used in this study.**

| Antibody/Reagent | Source | Identifier |
| --- | --- | --- |
| AKT | CST | 9272 |
| pAkt(S473) | SAB | 13357 |
| GSK-3β | SAB | 38353 |
| pGSK-3β(T216) | Upstate | 32160702 |
| pGSK-3β(S9) | CST | D2Y9Y |
| AT8 | ThermoFisher | MN1020 |
| Tau5 | Abcam | ab80579 |
| pT231-tau | SAB | 13381 |
| pS404-tau | SAB | 11112 |
| pT396-tau | Abcam | ab32057 |
| pT199-tau | Invitrogen | 44734G |
| pT181-tau | SAB | 11107 |
| pS262-tau | Abcam | ab80579 |
| pT217-tau | SAB | 11724 |
| IBA1 | Santa Cruz | 1022-5 |
| GFAP | CST | 3670 |
| IL-6 | Bio-techne | AF-406-NA |
| TNF-α | SAB | 41504 |
| Syn-1 | CST | D12G5 |
| SYP | SAB | 41475 |
| GluN1 | ABclonal | A7677 |
| GluR2 | ABclonal | A11316 |
| NeuN | CST | 24307 |
| β-actin | SAB | 21800 |
| HRP-conjugated goat anti-mouse IgG | Beyotime | A0216 |
| HRP-conjugated goat anti-rabbit IgG | Beyotime | A0208 |
| ECL luminol reagent | Beyotime | P0018FS |
| Streptozotocin(STZ) | Sigma | 572201 |
| Sodium citrate | Aladdin | 68-04-2 |
| Citric acid | Hushi | 10007108 |
| 9-ING-41(Elraglusib) | MCE | HY-113914 |
| PEG-400 | Solarbio | P8530 |

Antibody dilutions: 1:200 for immunostaining; 1:1000 for Western blotting.
